# Leveraging Deep Learning to Address Diagnostic Challenges with Insufficient Image Data

**DOI:** 10.1021/acssensors.5c01439

**Published:** 2025-09-11

**Authors:** Jian-Ming Lu, Ping-Yeh Chiu, Chien-Fu Chen

**Affiliations:** † Institute of Applied Mechanics, 33561 National Taiwan University, Taipei 106, Taiwan; ‡ Department of Orthopaedic Surgery, Chang Gung Memorial Hospital and Chang Gung University College of Medicine, Taoyuan 333, Taiwan; § Graduate School of Advanced Technology, 33561 National Taiwan University, Taipei 106, Taiwan

**Keywords:** generative adversarial networks, lateral flow immunoassay test, artificial intelligence, point of care, data augmentation

## Abstract

In recent AI-driven disease diagnosis, the success of models has depended mainly on extensive data sets and advanced algorithms. However, creating traditional data sets for rare or emerging diseases presents significant challenges. To address this issue, this study introduces a direct-self-attention Wasserstein generative adversarial network (DSAWGAN) designed to improve diagnostic capabilities in infectious diseases with limited data availability. DSAWGAN enhances convergence speed, stability, and image quality by integrating attention modules and leveraging the Wasserstein distance optimization. We compared DSAWGAN-generated images with traditional data augmentation and other image generation techniques, evaluating their effectiveness using classification neural networks for diagnostic accuracy. This model integration was then applied to a mobile app, enabling rapid, portable, and cost-effective diagnostic testing across various concentration ranges. Using only half of the raw data (*n* = 1500), DSAWGAN achieves an accuracy increase from 98.00 to 99.33%. Even with just 10% of the original data (*n* = 300), a neural network trained with the augmented data set maintains an accuracy of 92.67%, demonstrating the approach’s effectiveness in resource-limited settings.

## Graphical abstract

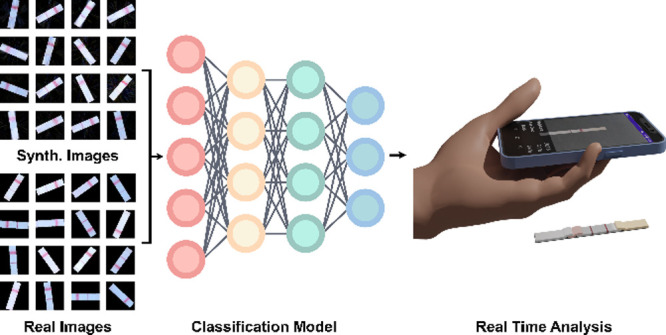

The lateral flow immunoassay (LFIA) has become a widely used diagnostic tool, particularly valued for its rapid and portable nature, making it ideal for point-of-care applications. Its ability to provide immediate results without extensive laboratory infrastructure has made it indispensable in various medical and public health settings.^1–3^ However, despite these advantages, the traditional interpretation of LFIA results remains limited by several key challenges. Visual inspection of the test results is primarily qualitative, providing limited information on precise concentration levels, which complicates the direct assessment of concentration ranges^4,5^ This shortfall often leads to the need for additional verification using specialized instruments or alternate methods, adding to the overall cost and procedural complexity. Moreover, recent studies have demonstrated that less experienced users can introduce variability in interpretation, yielding accuracy rates between 80 and 97% for distinguishing positive from negative samples.^6^

Integrating artificial intelligence (AI) into diagnostic testing addresses these challenges and drives substantial improvements in accuracy and efficiency, especially for rapid and portable diagnostic solutions. Deep learning techniques can process complex data, enhance diagnostic precision, and reduce interpretation times, which is crucial for real-time, point-of-care use.^7–15^ For example, smartphone-based AI solutions, such as deep learning-assisted LFIA (SMARTAI-LFA), have achieved 98% accuracy in blind tests across 1500 clinical samples, outperforming untrained individuals and trained experts.^16^ Additionally, time-series deep learning and AI approaches have significantly reduced LFIA result analysis time to just 2 min, compared to 15 min with human interpretation.^17^ In rural South Africa, these advancements have shown promising outcomes for HIV test image classification, achieving 97.8% sensitivity and 100% specificity, thereby supporting the development of affordable, real-time diagnostics accessible in resource-limited settings.^18^

During large-scale infectious disease outbreaks, rapidly gathering and analyzing substantial amounts of diagnostic data is essential for timely and effective response.^19,20^ However, the limitations of traditional diagnostic approaches, often requiring manual interpretation or specialized equipment, make it challenging to scale data collection in outbreak settings.^21^ This slows the tracking of infection spread and poses a significant challenge for artificial intelligence (AI) integration. Without a steady influx of high-quality, real-time data, AI algorithms struggle to adapt and optimize their models for accurate diagnostics, limiting their potential to support swift, data-driven decision-making in high-stakes situations.^22^ The need for up-to-date data can prevent AI from fully leveraging its predictive capabilities, hindering the rapid adjustments necessary to improve diagnostic accuracy, resource allocation, and containment strategies during critical early stages of an outbreak.^23^

While ample data sets exist for well-established LFIA applications such as COVID-19, influenza, and pregnancy tests, many less common or emerging diagnostic scenarios suffer from a scarcity of large-scale image collections. For diseases beyond mainstream commercial tests, publicly available LFIA image data sets are often limited or nonexistent. In research and development contexts, generating extensive LFIA data sets can be costly and time-consuming, particularly when targeting novel biomarkers, rare conditions, or limited patient cohorts.^24–27^ Moreover, the likelihood of future outbreaks of emerging large-scale infectious diseases is expected to increase due to factors such as global population growth, intensified international travel, climate change, and the expansion of zoonotic spillover events.^28–33^ These scenarios demand rapid LFIA development and deployment before sufficient real-world image data can be accumulated.

This study introduces a direct-self-attention Wasserstein generative adversarial network (DSAWGAN) to address these challenges and improve diagnostic efficacy in data-scarce settings. Unlike traditional GANs, DSAWGAN optimizes data generation by streamlining attention mechanisms, removing data reshaping, and preserving essential information. This architecture employs convolutional layers to extract features (Query, Key, and Value) and utilizes softmax normalization to create an attention map that selectively focuses on relevant data regions. Matrix multiplication of these features generates a detailed representation of complex relationships within the data, enhancing computational efficiency and interpretability.

Our multifaceted approach includes a robust data set organization strategy. We categorize data into several concentration labels, applying various augmentation techniques to simulate diverse scenarios for neural network training. Additionally, we validate model performance using confusion matrices,^34,35^ ROC curves,^36,37^ and dimensionality reduction techniques such as t-SNE.^38,39^ Integrating the DSAWGAN-generated data with a classification neural network, we achieve rapid, stable convergence and mitigate common issues like overfitting and instability in small data sets.

By integrating DSAWGAN in a mobile application, we further demonstrate the potential for rapid, accurate, cost-effective diagnostics suitable for low-resource environments. Experimental results show that the DSAWGAN architecture improves diagnostic accuracy, reaching 99.33% with only half of the original data set. It achieves 92.67% accuracy even with 10% of the data, highlighting its effectiveness in facilitating resource-efficient diagnostics.

## Experimental Section

### Reagents and Equipment

Nitrocellulose membrane (CN140) was purchased from Sartorius Stedim Biotech GmbH (Goettingen, Germany). The sample pad, conjugate pad, absorbent pad, adhesive backing card, and stacking pads were purchased from ShangHai KinBio Co. Ltd. (Shanghai, China). Bovine serum albumin (BSA), phosphate buffered saline (PBS, pH 7.4), phosphate buffered saline containing TWEEN 20 (PBS-T, pH 7.4), Protein A soluble from *Staphylococcus aureus*, Anti-Protein A produced in Rabbit, Anti-Rabbit IgG produced in Goat, were purchased from Sigma-Aldrich (St. Louis, MO, USA).

### Lateral Flow Immunoassay Manufacturing Process

The lateral flow immunoassay utilizes a test strip composed of a sample pad, conjugate pad, test pad, and absorbent pad. The sample undergoes sequential reactions with these regions through capillary action.^40^

First, the sample pad, made of materials such as glass fiber or cellulose, is the initial contact area with the specimen. Its function involves preliminary sample filtration to prevent matrix effects and stabilize the specimen. If the specimen contains the antibodies to be detected, they will bind to enzymes or nanomaterial-modified antibodies initially dried on the conjugate pad for subsequent reactions.

The test pad, consisting of a nitrocellulose membrane with a surface containing a highly protein-affinitive polyester film, is premarked with two lines: the test line and control line. The test line has specific antibodies capable of capturing the target analyte, while the control line captures antibodies labeled with nanomaterials, resulting in a signal for color development. The excess liquid is absorbed by the absorbent pad through capillary forces, stabilizing the flow field.

The complete lateral flow immunoassay testing process involves adding 20 μL of the sample to 150 μL of buffer solution (5% BSA in PBST) and conducting real-time detection through the lateral flow immunoassay test strip. The standard testing procedure requires a waiting period of 15 min to obtain the test results.

The sample pad and conjugate pad used in this experiment were predried with 5% BSA in PBST and AuNPs @ rabbit-antiprotein A, respectively. The test pad was marked with rabbit antiprotein for the test line and goat-antirabbit IgG for the control line. All drying processes were conducted at 37 °C for 2 h on a heating pad. Assembly of the test strip components, including sample pad, conjugate pad, test pad, and absorbent pad, onto a dedicated backing board completed the assembly process.

### Data for Deep Learning Models

In this study, samples from the lateral flow immunoassay test strips were categorized into three classes based on the concentration: negative, low concentration, and high concentration. The low concentration category includes concentrations of 0.5 and 0.25 μg/mL, while the high concentration category included concentrations of 1 and 0.75 μg/mL. To simulate diverse real-world testing scenarios, we introduced light sources from different directions during image capture, resulting in a total of 3000 original images of the lateral flow immunoassay test strips, with 1000 images per category (Table S1).

During the data preprocessing, we initially cropped the images based on the edges of the original data and standardized the resolution to 3 × 660 × 50. Subsequently, zero-padding was applied on both sides of the images to make them 3 × 660 × 660 in size. Then, we performed cropping based on the positions of the test line and control line, finally standardizing the images to 3 × 128 × 128. To further enhance data diversity, we chose to use geometric data augmentation through rotating the images by 3.6° in each iteration, totaling 360° rotation and resulting in a data set of 300,000 images.

### GANs and Augmentation for Diverse Data Sets

To address the time and cost associated with collecting large-scale LFIA data sets, this study employed generative adversarial networks (GANs) for synthetic training data generation, combined with data augmentation to form a mixed data set. Four GAN architectures were compared DCGAN, SAGAN, WGAN, and the proposed DSAWGAN selected to provide a balance between architectural diversity, historical significance, and computational feasibility. DCGAN employs a straightforward convolutional backbone suitable for low-complexity image synthesis; WGAN introduces the Wasserstein loss to improve training stability; and SAGAN incorporates self-attention mechanisms to capture long-range dependencies. These foundational design principles are directly related to the components integrated into DSAWGAN.^41–46^ Additionally, the selected architectures are computationally lightweight and can be trained effectively with limited resources, aligning with our long-term goal of enabling GAN-based image synthesis in resource-constrained environments such as mobile devices or edge computing platforms for field diagnostics.^47–49^

For quality control of generated data, we employed the Structural Similarity Index (SSIM) and Hamming distance to assess similarity levels, filtering out highly similar outputs to ensure data set diversity. The preprocessing pipeline for synthetic data was identical to that for real data. Following training, the GANs generated 150,000 images, which were then combined with the original real data set (*n* = 150,000) to form a mixed data set of 300,000 images.

### Fusion of Real and Synthetic Data for Method Validation

To validate the feasibility of the method, we combined real and synthetic data into two different types of training data: 100% real data and mixed data composed of 50% real data and 50% synthesized data. Each data type included five different quantities of image data, specifically 15,000, 30,000, 60,000, 150,000, and 300,000 image data. To assess the performance of the classification neural network, we also prepared an additional 600 original data images without data augmentation processing as a testing set.

### Parameters of Classification Neural Network

The ultimate goal of this study was to apply the synthesized data mixed with real data to a mobile-based image recognition system. Therefore, we selected classification neural network models suitable for compression into a mobile operating environment, including ResNet50, MobileNetV2, and EfficientNet B0. For the classification training of different concentrations of real lateral flow immunoassay images, in order to understand the strengths and weaknesses of different classification neural network architectures, we uniformly set the batch size to 32, optimizer to Adam, loss function to Cross Entropy Loss, and the number of training epochs to 100. We then examined, under the same training conditions, which model exhibited the best convergence and accuracy.

### Efficient Training with DSAWGAN Optimization

While SAGAN addresses common issues in neural networks such as gradient explosion, gradient vanishing, and overfitting, its practical training requires significant computational resources. This is due to the multiple transformations performed on the feature space during calculations, such as Reshape and Transpose. Therefore, we propose a training approach that avoids reshaping the feature space and directly computes the attention map. The advantage of this method lies in reducing training time substantially, saving unnecessary data type conversions, and obtaining a more intuitive attention map with spatial information.

Additionally, we incorporated the Wasserstein distance from WGAN to optimize the neural network, defined as follows.^50^ This further enhances the training process and improves the overall performance of the model.

$$W(P_{r},P_{g})\;=\underset{\gamma \in \prod (P_{r},P_{g})}{\inf}E_{(X,Y)∼\gamma }[∥X-Y∥]$$ 1

The term Wasserstein distance is derived from the interpretation that it measures the total amount of probability mass that needs to be transported to transform one distribution **P** *_r_* into another distribution **P** *_g_*. More formally, it considers the set of joint distributions that have **P** *_r_* and **P** *_g_* as marginal, and the Wasserstein distance is defined as the minimum transport cost required to achieve this transformation.

The optimization of the neural network is achieved by computing the minimum estimate of the expected distance between *x* and *y* in the distribution. The Attention calculation involves extracting image features from the previous hidden layer *x* ∈ R^*C*×*H*×*W*^. Initially, a 1 × 1 convolutional layer is used to extract the feature matrix, which is then transformed into two feature spaces ***Q*** and ***K*** for calculating Attention values. Here ***Q*** = ***f*** (***x***) = ***W*_*f*_ *x***, ***K*** = ***g*** (***x***) = ***W*_*g*_ *x***, and through softmax, the attention map is obtained. Subsequently, a matrix product is performed with the feature space ***V***, where ***V*** = ***v*** (***x***) = ***W*_*v*_ *x***. This results in the final feature space with attention-weighted values, and the computation process is outlined below.

$$\mathrm{Attention}(Q,K,V)=\mathrm{soft}\max(f(x_{i}{)}^{T}g(x_{i}))v(x_{i})$$ 2

Here ***W*_*f*_** ∈ **R** ^***C*** × ***H*** × ***W***^, ***W*_*g*_** ∈ **R** ^***C*** × ***H*** × ***W***^ and ***W*_*v*_** ∈ **R** ^***C*** × ***H*** × ***W***^ Additionally, we introduce a weight coefficient γ for the attention features, which adjusts during training to optimize the neural network’s feature extraction results.

$$y_{i}=\gamma o_{i}+x_{i}$$ 3

In the above equation, γ starts with an initial value of 0 and is adjusted based on the training results. Here, ***x*_*i*_** represents the feature space obtained from the previous hidden layer, ***o*_*i*_** is the output of the attention mechanism for the current training, and ***y*_*i*_** is the feature space obtained during the current training. This process aims to achieve a more stable feature output during the deep learning training, thereby accelerating the convergence of the neural network.

### Thorough Evaluation of Classifier Performance in Testing

This study investigated four performance metrics, including sensitivity, specificity, positive predictive value (PPV), and negative predictive value (NPV). For each image, the results generated by the classifier can be categorized into one of four classes: TP (true positive), TN (true negative), FP (false positive), or FN (false negative).^51^ The accuracy of the results depends on the comparison with the chosen gold standard.

Sensitivity is a metric that measures the classifier’s ability to correctly detect positive results. It is calculated as

$$\mathrm{Sensitivity}=\frac{\mathrm{TP}}{\mathrm{TP}+\mathrm{FN}}$$ 4

Specificity measures the classifier’s ability to correctly detect negative results. It is calculated as

$$\mathrm{Specificity}=\frac{\mathrm{TN}}{\mathrm{TN}+\mathrm{FP}}$$ 5

These metrics represent the proportions of true positives and true negatives among the confirmed positive and negative results in a diagnostic test.

In our study, we employed a variety of evaluation metrics to thoroughly assess the performance of the classification neural network. The confusion matrix, which delves into true positives, true negatives, false positives, and false negatives, allowed for a detailed examination of the model’s accuracy and misclassification tendencies across different classes. This analysis revealed that our classification neural network performed well, exhibiting higher counts of true positives and true negatives, coupled with relatively lower counts of false positives and false negatives. These results underscore the reliability of our model in the classification task.

Furthermore, we utilized t-SNE, a dimensionality reduction and visualization technique, to transform the feature representations extracted by the classification neural network into a lower-dimensional space. This visualization highlighted distinct separation and clustering patterns among different classes, demonstrating the model’s effectiveness in learning and distinguishing various features. Additionally, the evaluation of ROC curves, with AUC values approaching 1, indicated excellent performance across different thresholds. These comprehensive evaluations provide a nuanced understanding of the classifier’s capabilities, affirming its suitability and reliability for lateral flow immunoassay test sample detection and aiding decision-making in practical applications.

### Mobile Application

We developed an Android mobile application utilizing a trained EfficientNet B0 neural network for rapid diagnostic testing. The app features user-friendly design and portability. Training was conducted with a batch size of 32, input image dimensions of 128 × 128 × 3. and the Adam optimizer (learning rate: 0.0001) over 100 epochs.

To evaluate performance, we tested the app on three smartphones with varying models, brands, and Android versions: Sony Z5 (released in 2015, Android 7.1) with a Qualcomm Snapdragon 810 CPU, 3 GB of RAM, and a 23 MP camera; the Redmi Note 6 Pro (released in 2018, Android 8.1) with a Qualcomm Snapdragon 636 CPU, 4 GB of RAM, and a 12 MP camera; and the Asus Zenfone 5Z (released in 2018, Android 10) with a Qualcomm Snapdragon 845 CPU, 6 GB of RAM, and a 12 MP camera. TensorFlow Lite and Android Studio were used for model deployment and app development.

In testing, 30 lateral flow immunoassay strips were divided into high concentration (*n* = 10), low concentration (*n* = 10), and negative (*n* = 10) groups. The app demonstrated accurate and consistent detection across all devices, regardless of RAM, CPU, or Android version.

## Results and Discussion

Workflow and Experimental Design. Figure 1 outlines the multistage process of training a neural network on a small data set, including data preprocessing, data generation, and classification model validation, ensuring a robust neural network for mobile point-of-care applications.

**Figure 1:**
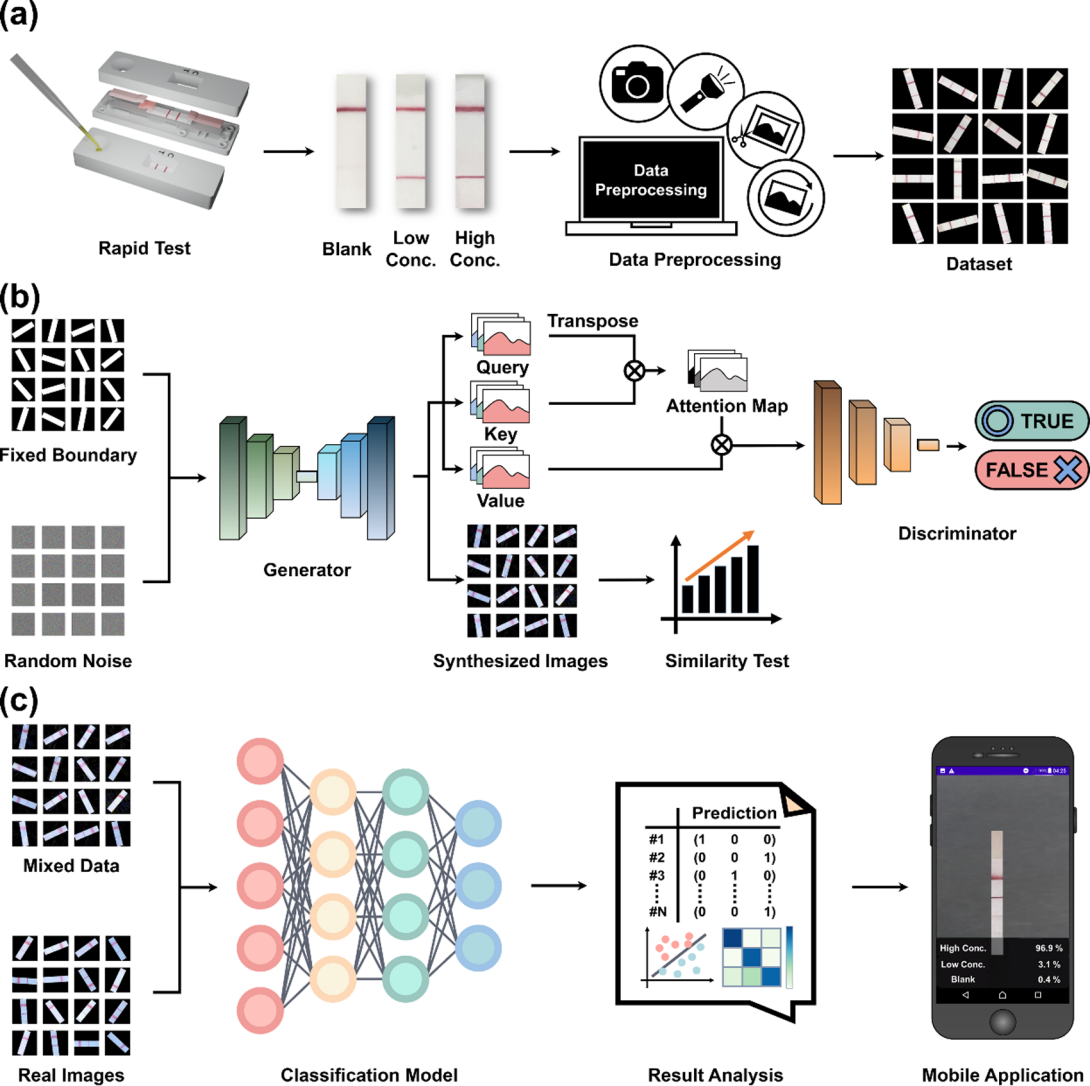
Experiment design using a generative adversarial network to address data collection challenges. (a) Standard image data collection process: The experiment begins with the acquisition of original images (*n* = 3000) in the data preprocessing stage, resulting in a real data set (*n* = 300,000) after the processing. (b) Synthetic data generation with DSAWGAN: The synthetic data generation process utilizes our designed DSAWGAN. It employs the structural similarity index (SSIM) and Hamming distance to eliminate overly similar synthesized data. The synthesized data is then combined with real data in a 1:1 ratio to form a mixed data set. (c) Training classification neural networks: The real data set and mixed data set are separately used to train classification neural networks. The training results from both data sets are analyzed, demonstrating the feasibility of training neural network models using mixed data sets. Finally, the model is integrated into a mobile application for real-time detection.

In stage one (Figure 1a), real images of lateral flow immunoassay strips detecting varying sample concentrations are collected under different lighting conditions to form a raw data set (*n* = 3000). This data set is expanded through augmentation techniques to *n* = 300,000, creating a diverse training base.

In stage two, Figure 1b presents the DSAWGAN workflow. Modifications to the standard GAN, such as introducing random noise, fixed boundary conditions, and U-Net architecture, enhance image quality and neural network stability. Inspired by WGAN, adjustments include removing the Sigmoid layer, using a logarithm-free loss function, and adopting RMSProp as the optimizer.^52^ The direct-self-attention module further optimizes recognition capabilities, ensuring high-quality data generation that outperforms traditional GANs.

In stage three (Figure 1c), ResNet50,^53^ MobileNetV2,^54^ and EfficientNet B0,^55^ are trained and evaluated on data sets of varying structures, with performance tested on real data. The optimal classification network is selected and integrated into a mobile app to accelerate the development of a mobile point-of-care system.

### Model Optimization for Concentration Classification

In order to select a suitable classification neural network, we created lateral flow immunoassay strips, analyzed test results, and utilized data augmentation techniques to establish different types of data (Figure 2a–c). These data sets were then applied to train classification neural networks (Figure 2d). Based on the training results (Table 1), it is evident that ResNet50, MobileNetV2, and EfficientNet B0 exhibited a converging trend in both loss function and accuracy calculations when trained on the real data set (*n* = 300,000). Additionally, evaluating the accuracy of classifying unknown data using a testing set (*n* = 600) was obtained through additional lateral flow immunoassay test results to ensure representativeness and reliability. The results shown in Figure 2e revealed that EfficientNet B0 achieved the highest accuracy (98.00%), followed by MobileNet V2 (96.50%), and ResNet 50 (80.83%), which performed the lowest.

**Figure 2:**
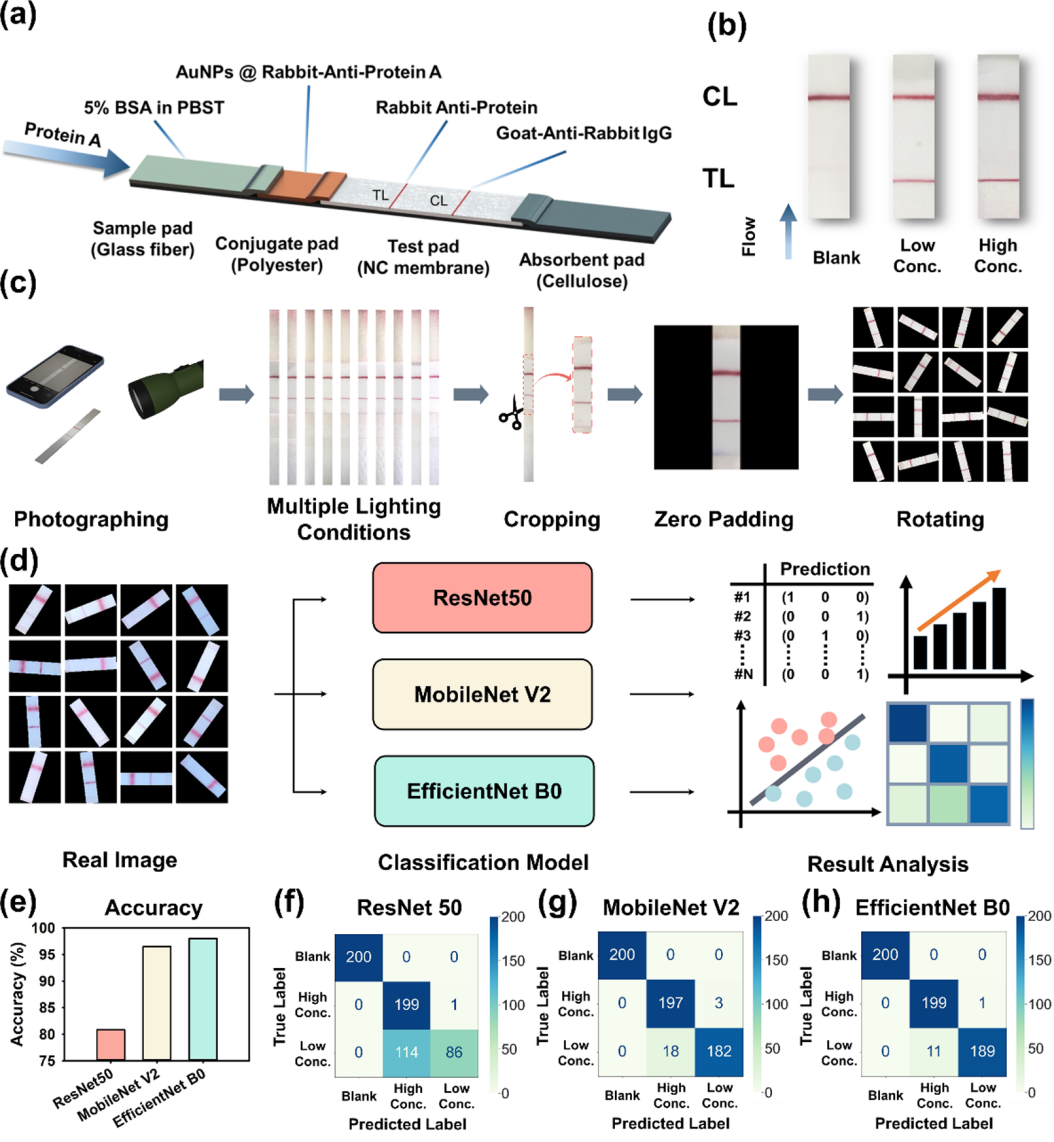
Real data preprocessing and classification neural network selection process. (a) Components of the lateral flow immunoassay chip. (b) Results from the lateral flow immunoassay chip are categorized into three labels, including high concentration (composed of concentrations 1 and 0.75 μg/mL), low concentration (composed of concentrations 0.5 and 0.25 μg/mL), and negative. (c) Data augmentation process. (d) Real data set, organized and processed, is input into ResNet 50, MobileNet V2, and EfficientNet B0 for classification training. The performance of neural network models is compared. (e) Classification neural network test results. (f–h) Confusion matrices results of each classification neural network.

**Table 1:** Classification Neural Network Test Results

		blank		high conc.		low conc.	
	acc.	SEN	SPE	SEN	SPE	SEN	SPE
ResNet50	0.8083	1.0000	1.0000	0.7150	0.9950	0.9975	0.4300
MobileNet V2	0.9650	1.0000	1.0000	0.9550	0.9850	0.9925	0.9100
EfficientNet B0	0.9800	1.0000	1.0000	0.9725	0.9950	0.9975	0.9450

Examining the confusion matrices for different classification neural networks on the testing set data (Figure 2f–h), it was observed that ResNet 50 struggled to effectively distinguish between low and high concentrations, often misclassifying low-concentration data as high-concentration. On the other hand, compared to the performance of MobileNet V2, the experimental results demonstrated that EfficientNet B0 exhibited superior sensitivity and specificity in classifying different concentrations. This superior performance can be attributed to the novel scaling method introduced in EfficientNet, which optimizes model depth, width, and resolution simultaneously, resulting in better representation learning across various scales of features.^56^ Moreover, the application of k-fold cross validation further validated EfficientNet B0s high stability across various subsets of the data set,^57,58^ confirming its robustness in handling classification tasks (Figure S1). Therefore, this study selected EfficientNet B0 as the classification neural network architecture for further comparison among different categories and magnitudes of data.

### Design and Comparison of Generative Adversarial Networks

The design and comparison of generative adversarial networks (GANs) were conducted in this study using a deep convolutional GAN (DCGAN),^59^ a Wasserstein GAN (WGAN), a self-attention GAN (SAGAN),^60^ and a direct-self-attention Wasserstein GAN (DSAWGAN) to generate a substantial amount of data from lateral flow immunoassay analysis data sets. To avoid generating duplicate data, we utilized Hamming distance^61–63^ and a structural similarity index (SSIM)^64,65^ to assess and filter the quality of data generated by different GANs, as well as to eliminate redundantly synthesized data (Figure 3a,b). Hamming distance was utilized to evaluate the similarity between data; a higher value indicating greater data dissimilarity. However, in some cases, such as failed image generation, using Hamming distance alone cannot effectively determine the completeness of the synthesized data structure. To ensure effective data quality control, SSIM was used to assess the structural similarity between generated and real data, with values ranging from 0 to 1. A higher value indicates less data dissimilarity, with 1 representing identical data structures.

**Figure 3:**
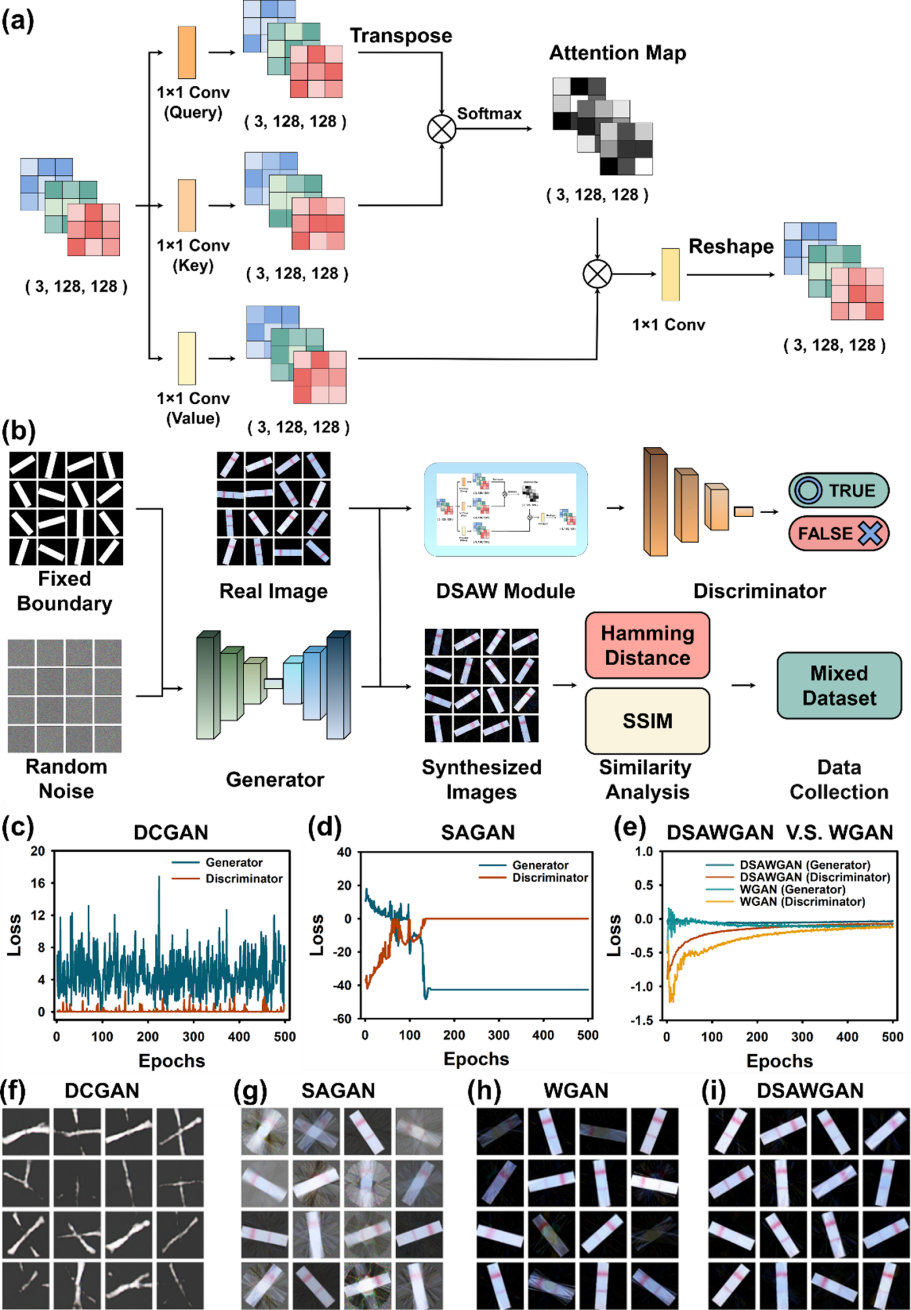
Operation process, design, and analysis of generative adversarial networks. (a) Direct–self–attention module workflow design. (b) Model architecture of DSAWGAN. (c–e) Convergence results of different generative adversarial networks. (f–i) Examples of synthetic data generated by different generative adversarial networks are illustrated.

As shown in Figure 3c–e, GANs are a common unsupervised learning model. Different GANs were employed to synthesize lateral flow immunoassay analysis data, and it was observed that the training processes of the DCGAN and SAGAN were unstable, leading to difficulties in convergence and incomplete image generation (Figure S2). We explored a comprehensive range of hyperparameter configurations in an attempt to stabilize these models, testing multiple learning rates (0.001, 0.0001, and 0.00001), batch sizes (16, 32, and 64), and optimization algorithms (Adam and RMSprop). Despite these efforts, both architectures consistently exhibited instability, including frequent mode collapse, failure to generate discernible LFIA-specific features, and unstable loss behavior (Figures S3–S6). These issues persisted across different experimental settings, indicating that the failure was not due to superficial parameter tuning but rather an architectural mismatch with the characteristics of LFIA data. On the other hand, both the WGAN and DSAWGAN exhibited converging trends in loss functions. Notably, the DSAWGAN showed faster convergence and better stability in generating high-quality images compared to the WGAN, making it suitable for data generation tasks (Figure 3f–i).

### Training Time and Stability Comparison Among Generative Adversarial Networks

In this study, we carefully evaluated the training time and stability of four GAN architectures DCGAN, WGAN, SAGAN, and the proposed DSAWGAN on lateral flow immunoassay (LFIA) data synthesis tasks (Table S2). Training time is a practical consideration in real-world applications, but it must be balanced against the quality and reliability of the generated data.

While DSAWGAN requires slightly more training time than WGAN (25,512 s versus 25,054 s), it offers significantly better training stability, which is critical for generating high-quality and reliable synthetic data. This trade-off is acceptable, particularly in applications where robustness and data quality are prioritized. Compared to DCGAN, which trains faster (12,004 s) but suffers from instability and poor performance in our experiments, DSAWGAN provides a balanced and practical solution. Although SAGAN exhibits a similar training time (27,075 s), it does not demonstrate the same level of stability and data quality as DSAWGAN.

### Comparison between WGAN and DSAWGAN

To quantitatively evaluate the performance differences between WGAN and the proposed DSAWGAN in the context of LFIA data generation, we conducted a downstream classification task using EfficientNet-B0 as the classifier. The synthesized images from both models were used to augment the training data set, and their impact on classification performance was assessed.

The evaluation included classification accuracy, confusion matrix analysis, and t-distributed stochastic neighbor embedding (t-SNE) visualizations. As in Figure S7 and Table S3, DSAWGAN-generated images consistently yielded higher classification accuracy compared to those produced by WGAN. Furthermore, the confusion matrix revealed fewer misclassifications, and the t-SNE plots showed clearer class separation for DSAWGAN-augmented data sets, indicating improved feature discriminability.

These results suggest that the architectural design of DSAWGAN, particularly the integration of direct self-attention mechanisms with the Wasserstein loss, facilitates the generation of more task-relevant and structurally coherent LFIA images. This enhanced image quality translates into superior utility for downstream analytical models, supporting the choice of DSAWGAN for subsequent data generation tasks in this study.

### Classification Performance of Neural Networks on Different Amount of Data

We aimed to prevent overfitting and validate the feasibility of training classification neural networks (CNNs) using mixed data. Data sets were organized with a 50% ratio of real to synthetic data, split into mixed and real data sets, with varying sizes (*n* = 15,000, 30,000, 60,000, 150,000, and 300,000). The hold-out method, with an 80:20 split ratio was applied for training and validation,^66,67^ and a testing set (*n* = 600) was prepared, consisting of 200 negative, 200 high-concentration (1 and 0.75 μg/mL), and 200 low-concentration (0.5 and 0.25 μg/mL) samples, and results are summarized in Table S4.

For data sets smaller than 60,000 (*n* < 60,000), both real and mixed data showed unstable performance, with testing accuracy below 80% (Figure 4e). At *n* = 60,000, while sensitivity and specificity exceeded 88% for negatives, performance for high and low concentrations remained inconsistent, with some values below 50%. When data set size increased to 150,000, accuracy, sensitivity, and specificity all exceeded 90%, and for *n* = 300,000, the real data set achieved 98% accuracy with 100% sensitivity and specificity for negatives, though specificity for low concentrations was slightly lower (94.5%). Training with mixed data (*n* = 150,000 real and 150,000 synthetic data generated by DSAWGAN) achieved superior performance, with an accuracy of 99.33% and sensitivity and specificity exceeding 99% across all classes (Table 2).

**Table 2:** Comparison of Classification Neural Network Training Results Using Real and Mixed Datasets^a^

		blank		high conc.		low conc.	
	acc.	SEN	SPE	SEN	SPE	SEN	SPE
real data	0.9800	1.0000	1.0000	0.9725	0.9950	0.9975	0.9450
mixed data	0.9933	0.9975	1.0000	0.9950	0.9900	0.9975	0.9900

a Evaluation of training results with test set data (*n* = 600) for real and mixed datasets.

**Figure 4:**
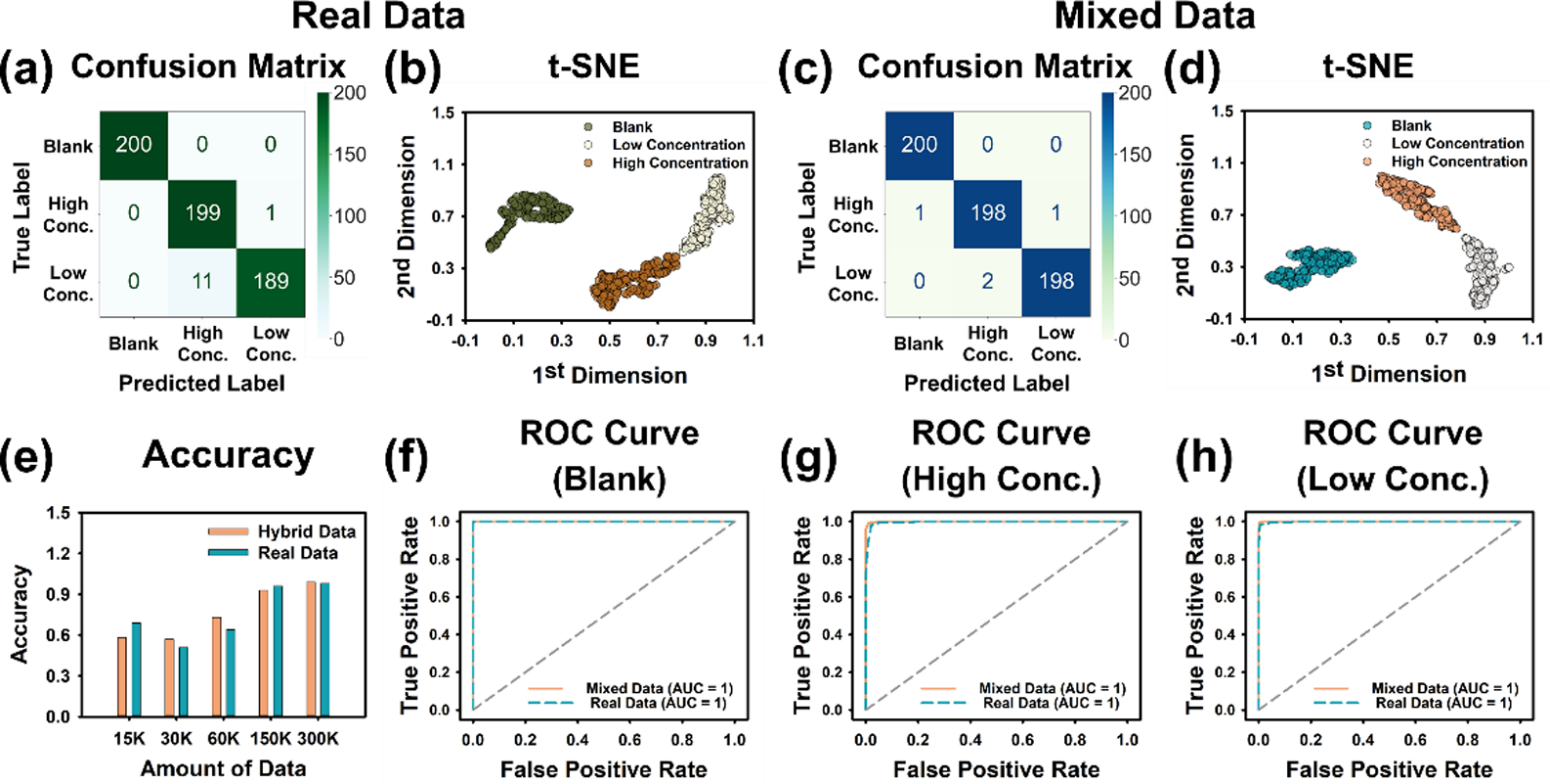
Comparison of classification neural network training results using real and mixed data sets. (a, b) Confusion matrix and t-SNE visualization for real data set training results on test set predictions. (c, d) Confusion matrix and t-SNE visualization for mixed data set training results on test set predictions. (e) Accuracy results of classification neural network training using real and mixed data sets with different quantities. (f–h) Roc curve analysis to examine consistency in classification results between real and mixed data sets.

We also utilized confusion matrices, t-SNE, and ROC curves to evaluate classification models and validate the feasibility of using mixed data sets. Confusion matrices (Figures 4a,c, S8A–E and S9A–E) reveal classification performance, showing TP, TN, FP, and FN. When training data quantity is under 60,000, models exhibit significant misclassifications, especially in high- and low-concentration categories. With 150,000 samples, classification improves, but errors persist (e.g., 15 misclassified low-concentration samples in real data, 19 in mixed data). At 300,000 samples, errors decrease significantly, with only 11 and 2 misclassified low-concentration samples for real and mixed data sets, respectively, highlighting the mixed data set’s superior performance. The t-SNE plots (Figures 4b,d, S8F–J and S9F–J) illustrate data distribution. At 150,000 samples, clearer class separation emerges, especially for negative data. At 300,000 samples, distinct clusters form, reflecting excellent classification. ROC curves (Figure 4f–h) further evaluate model performance, with AUC values near 1 for both real and mixed data sets, confirming robust classification.

This indicates that training with mixed data not only matches but, in some cases, surpasses results obtained with real data. Mixed data enriches feature diversity, mitigates sample scarcity, and enhances generalization, as evidenced by the high similarity between DSAWGAN-generated and real data. Moreover, using mixed data reduces the requirement for real data by half while achieving comparable performance, confirming its feasibility for high-performance classification model training.

### Superiority of Mixed Data Sets in Training Neural Networks

To compare the effectiveness of a mixed data set (real and synthesized data) with data augmentation, we adjusted the original data into binary label data (negative, positive) and multilabel data (negative, 0.25, 0.5, 0.75, and 1 μg/mL). Using the optimal parameters from previous experiments, we trained the EfficientNet B0 model for both binary and multilabel data.

The results show that training with a mixed data set outperforms data augmentation. For binary labels (Figure S10 and Table S5), both approaches achieved similar results, with 99.25% accuracy, 98.50% sensitivity and 100.00% specificity for negative labels, and 100.00% sensitivity and 98.50% specificity for positive labels, demonstrating the feasibility of the mixed data set approach.

For multilabel data (Figure S11 and Table S6), the mixed data set achieved 96.00% accuracy, with sensitivity and specificity ranging from 92.00 to 100.00%. In contrast, data augmentation resulted in only 93.60% accuracy, with sensitivity and specificity ranging from 82.00 to 100.00%. The mixed data set method significantly improved performance, especially for multilabel data.

### Limits of Overusing a Geometric Data Augmentation Method

Classical data augmentation typically involves geometric transformations, scaling, or adding noise to increase the size of the data set. To demonstrate the limited effectiveness of excessive data augmentation in improving the accuracy, sensitivity, and specificity of a classification neural network model, data sets with different processing methods were organized.^68–70^ Using half of the real data as prototypes, flipping was applied to generate 150,000 augmented samples, alongside 150,000 flipped samples. These were compared to a fully real data set (*n* = 300,000) and a mixed data set (150,000 augmented data and 150,000 generated by DSAWGAN). EfficientNet B0 was used consistently, with uniform training parameters.

The results (Figure S12 and Table S7) show that traditional data augmentation (rotation and flipping) performed comparably to geometric rotation alone (*n* = 300,000) during training but failed to improve classification performance on the test set. Accuracy was 52.00%, sensitivity ranged from 63.00 to 100.00%, and specificity ranged from 37.00 to 97.50%, indicating potential overfitting.

Visualization of results further highlights significant misclassifications, with high-concentration samples often classified as low concentration and low-concentration samples as negative. These findings demonstrate that excessive geometric data augmentation not only fails to enhance neural network training but may also lead to overfitting.

### Data Composition Impact on Training Results

To explore the minimum real data needed for effective training, we tested two data compositions: 10% real and 90% synthesized data (300 real, 2700 synthesized), and 1% real and 99% synthesized data (30 real, 2970 synthesized). These data sets were used to train an EfficientNet B0 classification neural network, with results shown in Figure S13 and Table S8.

When using 10% real and 90% synthesized data, the network achieved 92.67% accuracy, with sensitivity ranging from 91.75 to 99.75% and specificity from 88.00 to 100.00%. However, low-concentration samples were sometimes misclassified. In contrast, with 1% real and 99% synthesized data, accuracy dropped to 58.67%, sensitivity ranged from 68.75 to 91.00%, and specificity from 23.00 to 87.50%, indicating poor performance.

These findings emphasize the importance of real data proportions in GAN training. While a 10% real data composition supports stable and accurate performance, increasing the synthesized data to 99% significantly degrades the model’s ability to classify unknown data. A careful balance of real and synthesized data is crucial for ensuring reliable neural network training in practical applications.

### Real-Time Testing with a Smartphone Application

Table 3 outlines the simplified process of deploying the classification neural network in a mobile application. The model trained with mixed data was integrated into the app, enabling real-time image analysis on various smartphone models to assess its versatility.

**Table 3:** Real-Time Detection Results on Different Mobile Phones, Including Accuracy, Sensitivity, and Specificity for Classification of Various Label Data

		blank		high conc.		low conc.	
	acc.	SEN	SPE	SEN	SPE	SEN	SPE
Sony Z5	0.9333	1.0000	1.0000	0.9000	1.0000	1.0000	0.8000
Redmi Note 6 Pro	1.0000	1.0000	1.0000	1.0000	1.0000	1.0000	1.0000
Asus Zenfone 5Z	1.0000	1.0000	1.0000	1.0000	1.0000	1.0000	1.0000

The network analyzes input images and outputs predicted probabilities (summing to 1) for three labels, with higher probabilities indicating stronger classification confidence. Tables S9–S11 detail classification results for 30 data points across three smartphones, including predicted values, true labels, and network outputs.

Using 30 pretested lateral flow immunoassay strips (high concentration: *n* = 10, low concentration: *n* = 10, negative: *n* = 10), we conducted real-time analyses. Both Redmi Note 6 Pro and Asus Zenfone 5Z achieved perfect accuracy, sensitivity, and specificity (100%). However, the Sony Z5 exhibited recognition errors, misclassifying two low-concentration samples as high-concentration, resulting in 93.33% accuracy (Figure 5a,b). Confusion matrices (Figure 5c–e) highlight the generalizability of the classification neural network across different smartphone models.

**Figure 5:**
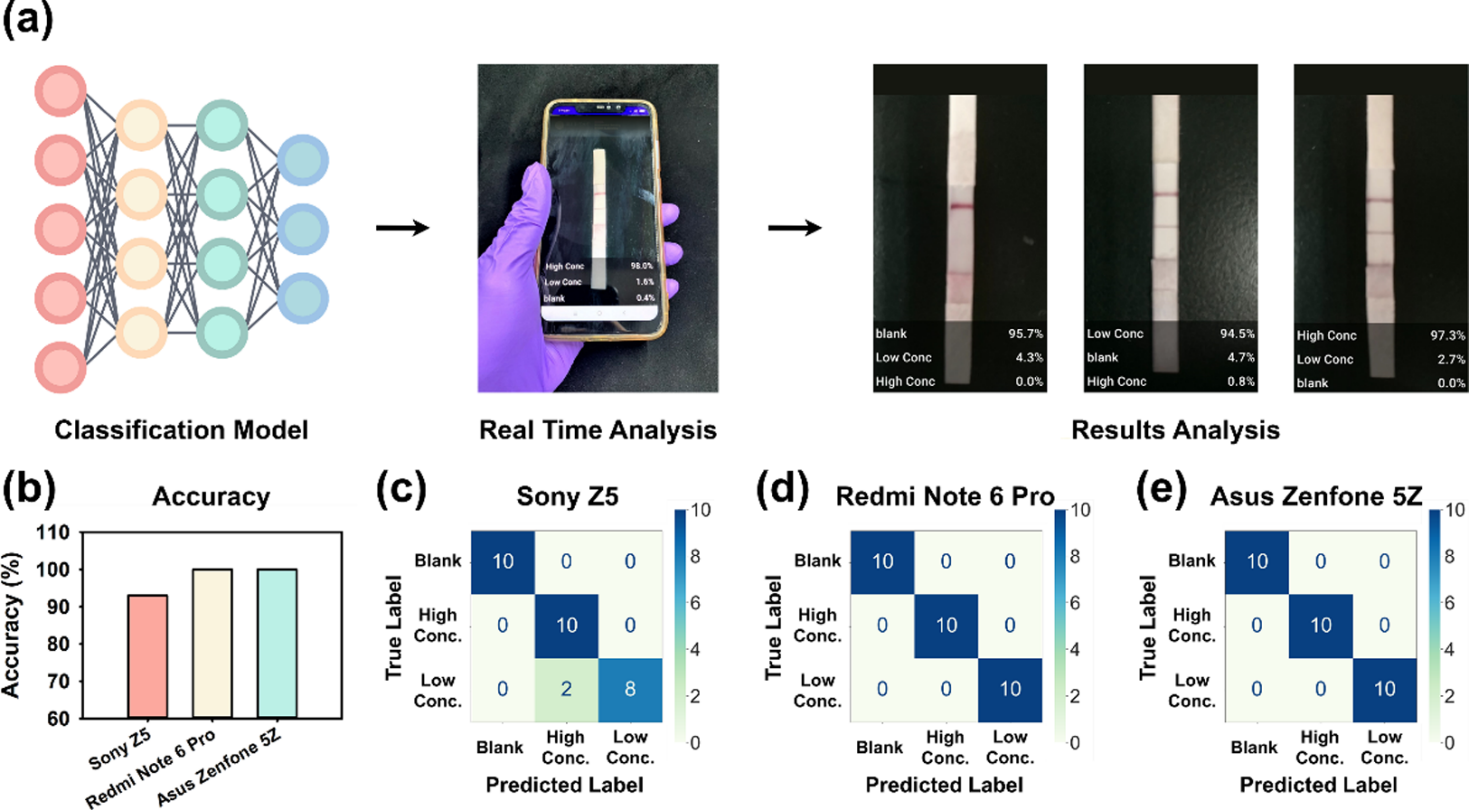
Real-time detection process using different mobile phone models and operating system versions, with results analysis to demonstrate universality. (a) Real-time detection process on mobile phones, integrating the trained classification neural network into a mobile application for immediate testing and verification of recognition results. (b) Comparison of accuracy in real-time recognition using different mobile phones. (c–e) Confusion matrices examining real-time detection results on different mobile phones.

### Validation with Clinical Sample

To validate the stability of the classification model, we conducted experiments using clinical human synovial fluid samples mixed with varying concentrations of Protein A solution (0.25, 0.5, 0.75, and 1 μg/mL) (Figure 6a). Classification neural networks trained with real data and mixed data were compared for their ability to classify these samples (Table 4 and Figure 6b,c).

**Table 4:** Results of Classification Neural Network Training Using Different Datasets

		blank		high conc.		low conc.	
	acc.	SEN	SPE	SEN	SPE	SEN	SPE
real data	0.9333	0.9500	1.0000	1.0000	0.8000	0.9500	1.0000
mixed data	0.9667	1.0000	1.0000	1.0000	0.9000	0.9500	1.0000

**Figure 6:**
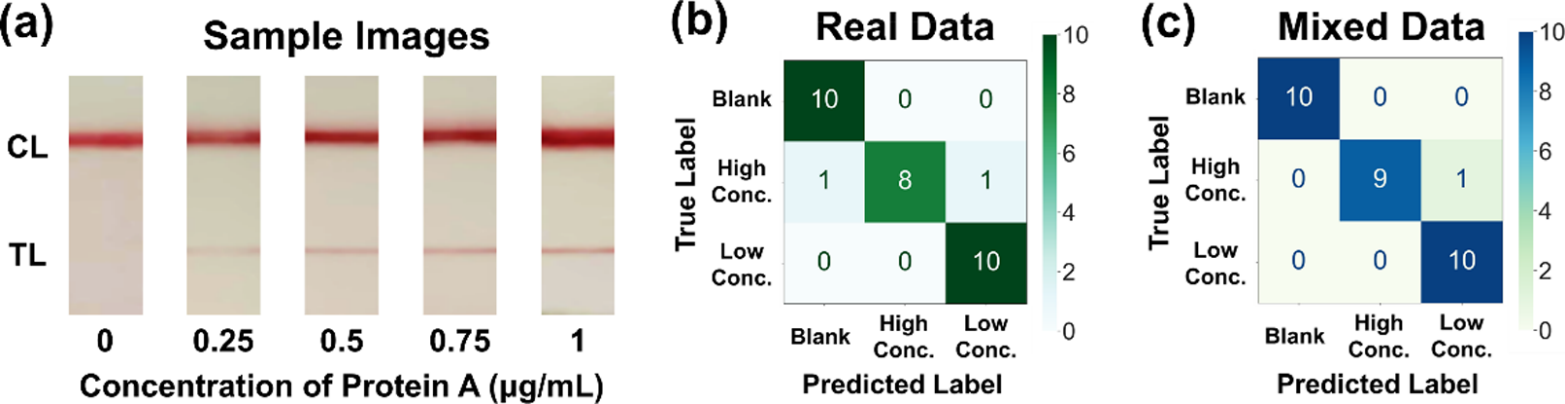
Testing and comparing classification neural network training using real and mixed data sets, applied to real samples. (a) Experimental results with real samples. (b) Confusion matrix representing test set classification results for a classification neural network trained with a real data set. (c) Confusion matrix representing test set classification results for a classification neural network trained with a mixed data set.

Thirty sets of lateral flow immunoassay test strips, including high concentration (*n* = 10), low concentration (*n* = 10), and negative (*n* = 10) samples, were tested. The network trained on real data achieved 93.33% accuracy, with sensitivity and specificity exceeding 95.00% for most classifications, except for slightly lower specificity (80.00%) for high concentration samples. In contrast, the network trained on mixed data achieved higher accuracy (96.67%), with sensitivity and specificity ranging from 90.00 to 100.00% across all classifications.

These results confirm the feasibility of using mixed data to train classification networks, achieving performance comparable to models trained on full real data while reducing the cost and time associated with data collection.

## Conclusions

This study presents an efficient method for training classification neural networks using small data sets, specifically designed for mobile point-of-care applications. By integrating data augmentation, DSAWGAN-generated synthetic data, and mainstream classification models such as ResNet50, MobileNetV2, and EfficientNet B0, the proposed approach achieves superior generalization capabilities and excellent performance. Notably, EfficientNet B0 achieved a test accuracy of 99.33% on a mixed data set, representing an improvement of approximately 1.33% over traditional augmentation techniques.

Experimental results demonstrate that the use of mixed data sets, combining real and synthetic data, significantly enhances the model’s ability to recognize unseen samples while reducing dependency on real data. This approach streamlines the development process, enabling the completion of robust neural network training within a few weeks, offering a cost-effective and time-efficient solution. Real-time testing with mobile applications highlights its high accuracy across different smartphone models, while validation with clinical samples further confirms the reliability and practicality of mixed data training.

Although this study focuses on LFIA as a use case, the proposed framework is modular and domain-agnostic. To adapt it to other diagnostic tasks with limited and costly imaging data, such as magnetic resonance imaging (MRI), computed tomography (CT), positron emission tomography (PET), or rare disease imaging, the deployment process involves replacing the LFIA-specific preprocessing pipeline with modality-appropriate preprocessing (e.g., normalization, denoising, or contrast enhancement), applying direct self-attention mechanisms within the generative model to produce high-quality synthetic images that enrich data diversity, and integrating the combined real and synthetic data sets into the training of classification neural networks. This approach not only addresses data scarcity but also enhances model robustness in imaging domains.

Our current clinical validation, based on 30 patient samples, serves as a proof-of-concept. We plan to include larger and more diverse cohorts in future work to improve generalizability. Further research will focus on applying this framework to other domains, fine-tuning model parameters to address smartphone variability, and expanding the data set to cover more pathogens and diagnostic scenarios. Integration with other mobile health tools may further extend its use in both clinical and environmental settings. Overall, this study offers a practical and scalable solution for building reliable mobile diagnostic systems.

## Supplementary Material



## Notes

Detailed training parameter settings and additional experimental results referenced in the main text (PDF)
