# Supplementary material for: Leveraging Deep Learning to Address Diagnostic Challenges with Insufficient Image Data
Source: ACS Sens. 2025 Sep 11;10(9):6734–45. doi: 10.1021/acssensors.5c01439 (PMC12481554; doi:10.1021/acssensors.5c01439)
Supplement: Supplementary file 1 [file se5c01439_si_001.pdf]

Supporting Information

Leveraging Deep Learning to Address Diagnostic Challenges with Insufficient Image Data

Jian-Ming Lu,[a] Ping-Yeh Chiu,[a, b] and Chien-Fu Chen[a, c*]

[a] Institute of Applied Mechanics, National Taiwan University, Taipei 106, Taiwan.

[b] Department of Orthopaedic Surgery, Chang Gung Memorial Hospital and Chang Gung University College of Medicine, Taoyuan 333, Taiwan

[c] Graduate School of Advanced Technology, National Taiwan University, Taipei 106, Taiwan

*Corresponding author: Chien-Fu Chen (stevechen@ntu.edu.tw)

Experimental results

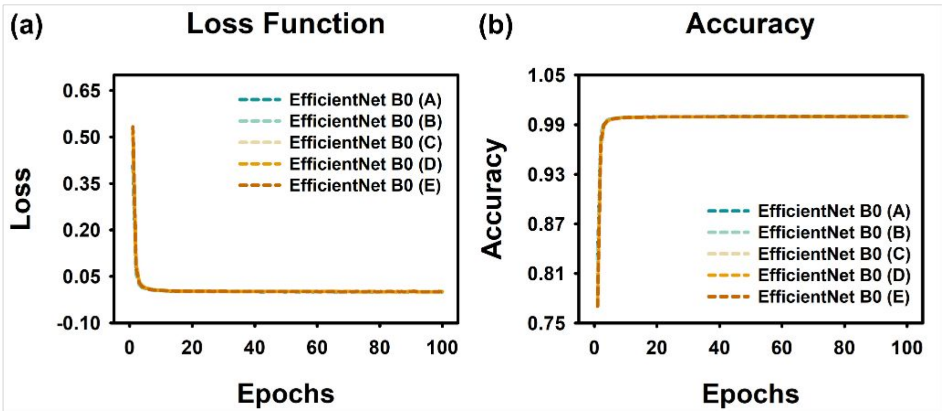

Fig. S1. Displaying the K-fold Cross Validation (k = 5) training process of EfficientNet B0 using real data. After 100 iterations, it can be observed in (**A**) and (**B**) that the results of the five training sessions converge and overlap, indicating the stability of the classification neural network and the training process with the dataset.

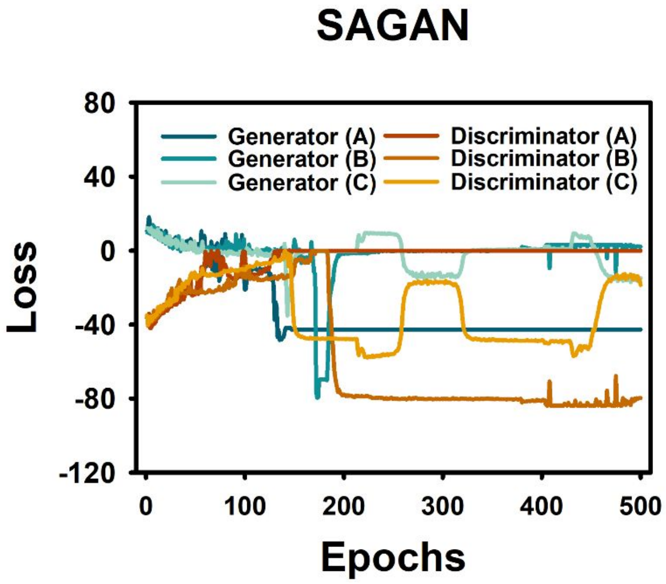

Fig. S2. Showcasing the training process of SAGAN in generating images. After 500 iterations, it can be observed that the results of three training sessions are all highly unstable. This indicates that SAGAN is not suitable for the generation task in this study.

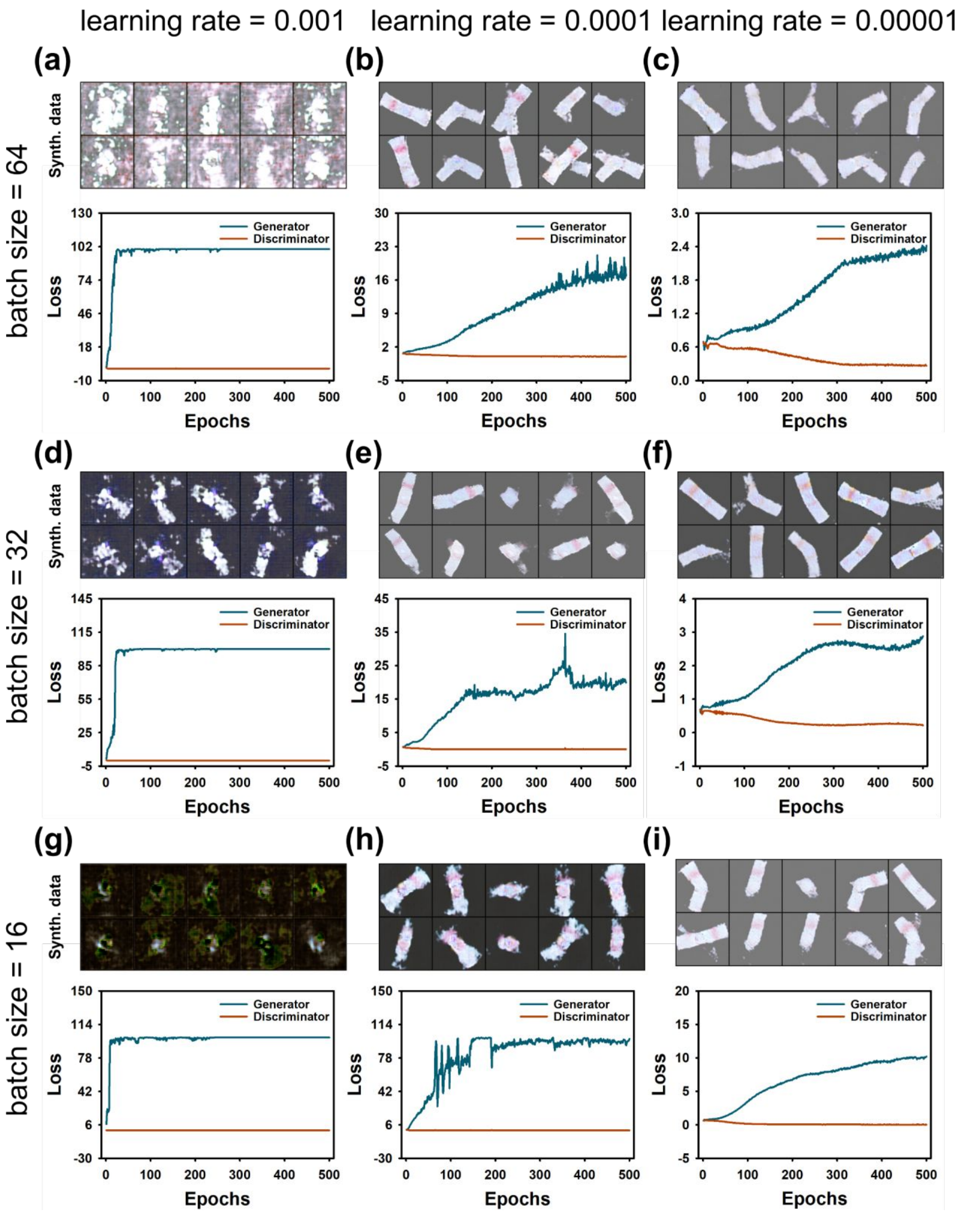

Fig. S3. Training stability analysis of DCGAN using the Adam optimizer, evaluated across different parameters: (a) learning rate = 0.001, batch size = 64; (b) learning rate = 0.0001, batch size = 64; (c) learning rate = 0.00001, batch size = 64; (d) learning rate = 0.001, batch size = 32; (e) learning rate = 0.0001, batch size = 32; (f) learning rate = 0.00001, batch size = 32; (g) learning rate = 0.001, batch size = 16; (h) learning rate = 0.0001, batch size = 16; (i) learning rate = 0.00001, batch size = 16.

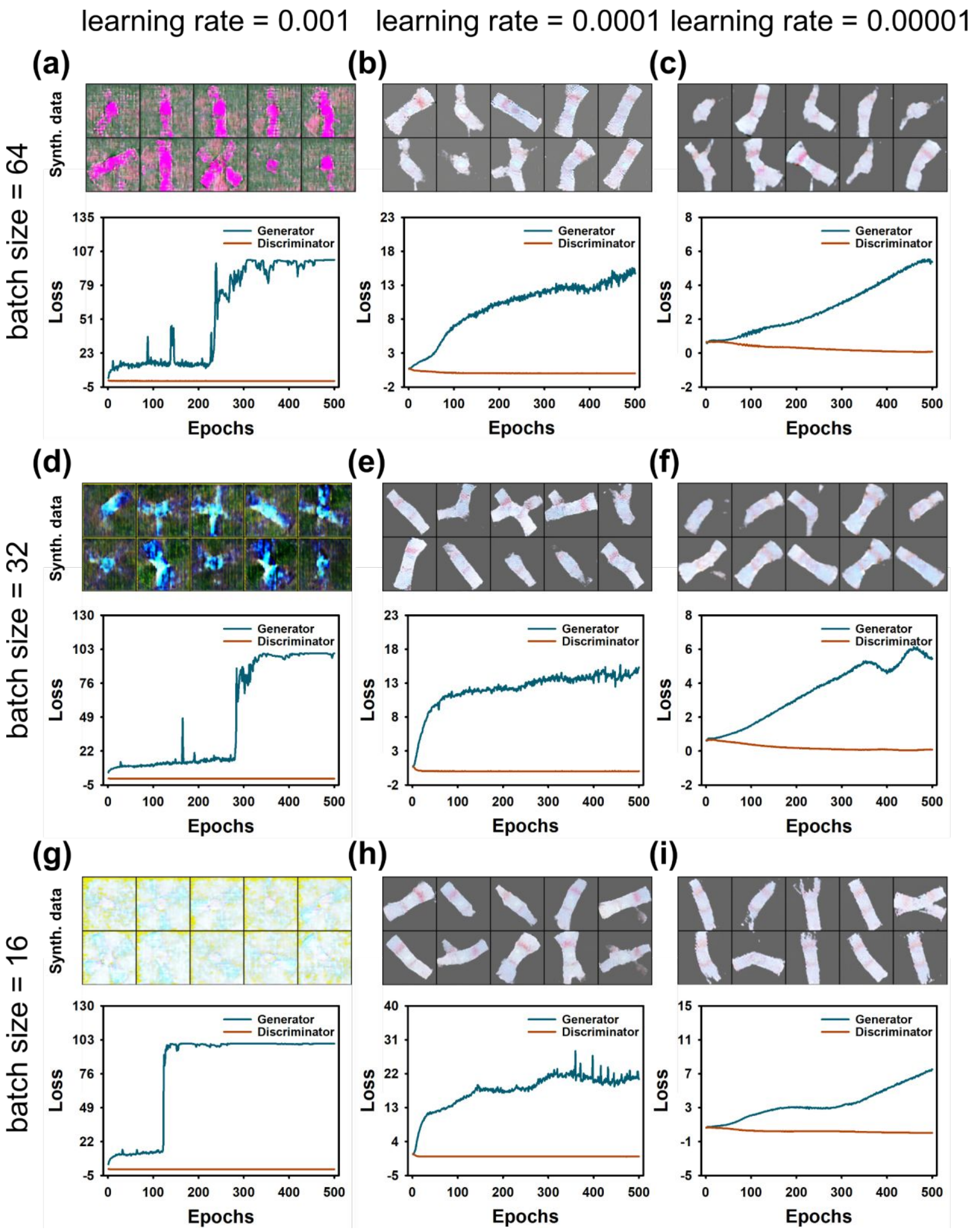

Fig. S4. Training stability analysis of DCGAN using the RMSprop optimizer, evaluated across different parameters: (a) learning rate = 0.001, batch size = 64; (b) learning rate = 0.0001, batch size = 64; (c) learning rate = 0.00001, batch size = 64; (d) learning rate = 0.001, batch size = 32; (e) learning rate = 0.0001, batch size = 32; (f) learning rate = 0.00001, batch size = 32; (g) learning rate = 0.001, batch size = 16; (h) learning rate = 0.0001, batch size = 16; (i) learning rate = 0.00001, batch size = 16.

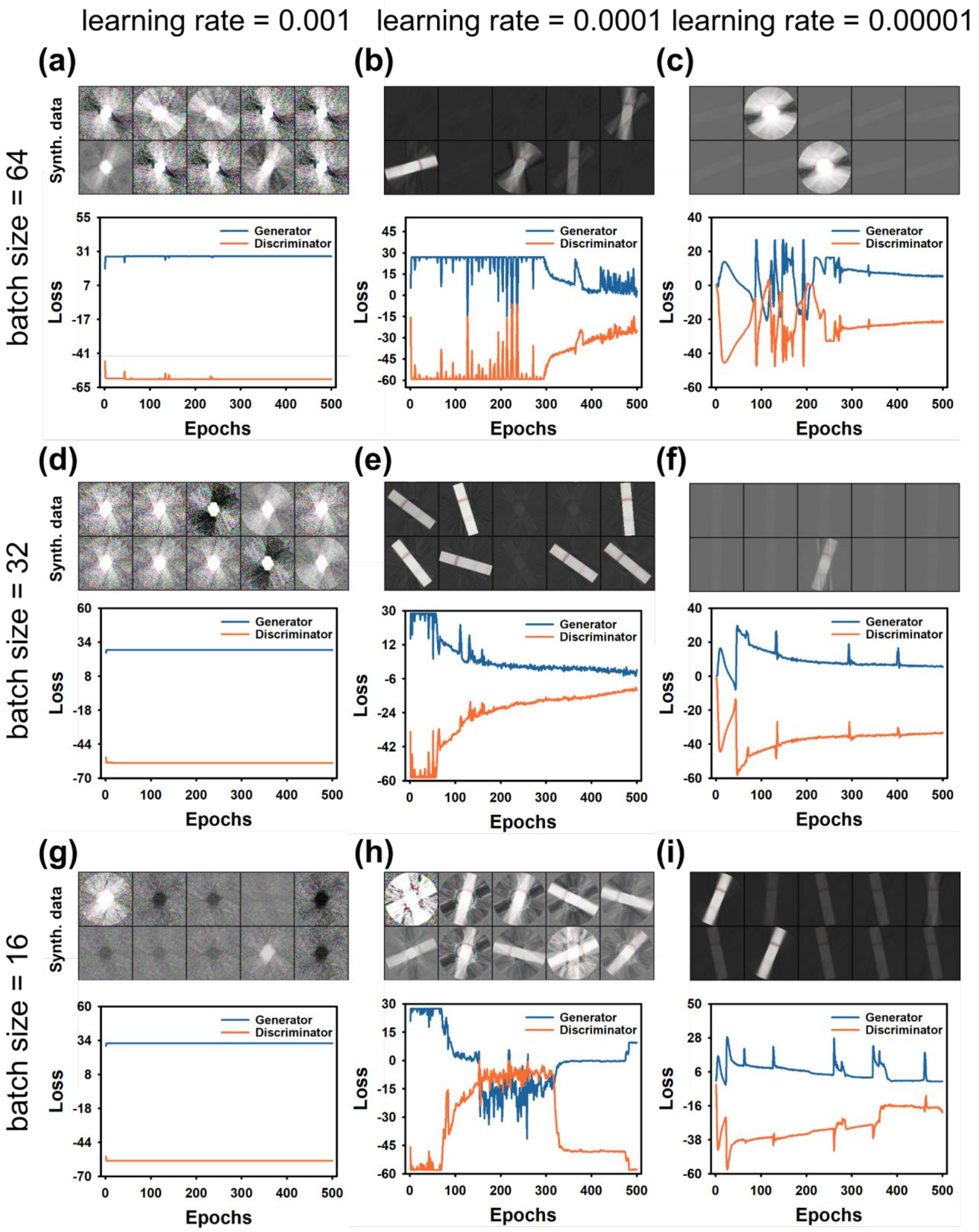

Fig. S5. Training stability analysis of SAGAN using the Adam optimizer, evaluated across different parameters: (a) learning rate = 0.001, batch size = 64; (b) learning rate = 0.0001, batch size = 64; (c) learning rate = 0.00001, batch size = 64; (d) learning rate = 0.001, batch size = 32; (e) learning rate = 0.0001, batch size = 32; (f) learning rate = 0.00001, batch size = 32; (g) learning rate = 0.001, batch size = 16; (h) learning rate = 0.0001, batch size = 16; (i) learning rate = 0.00001, batch size = 16.

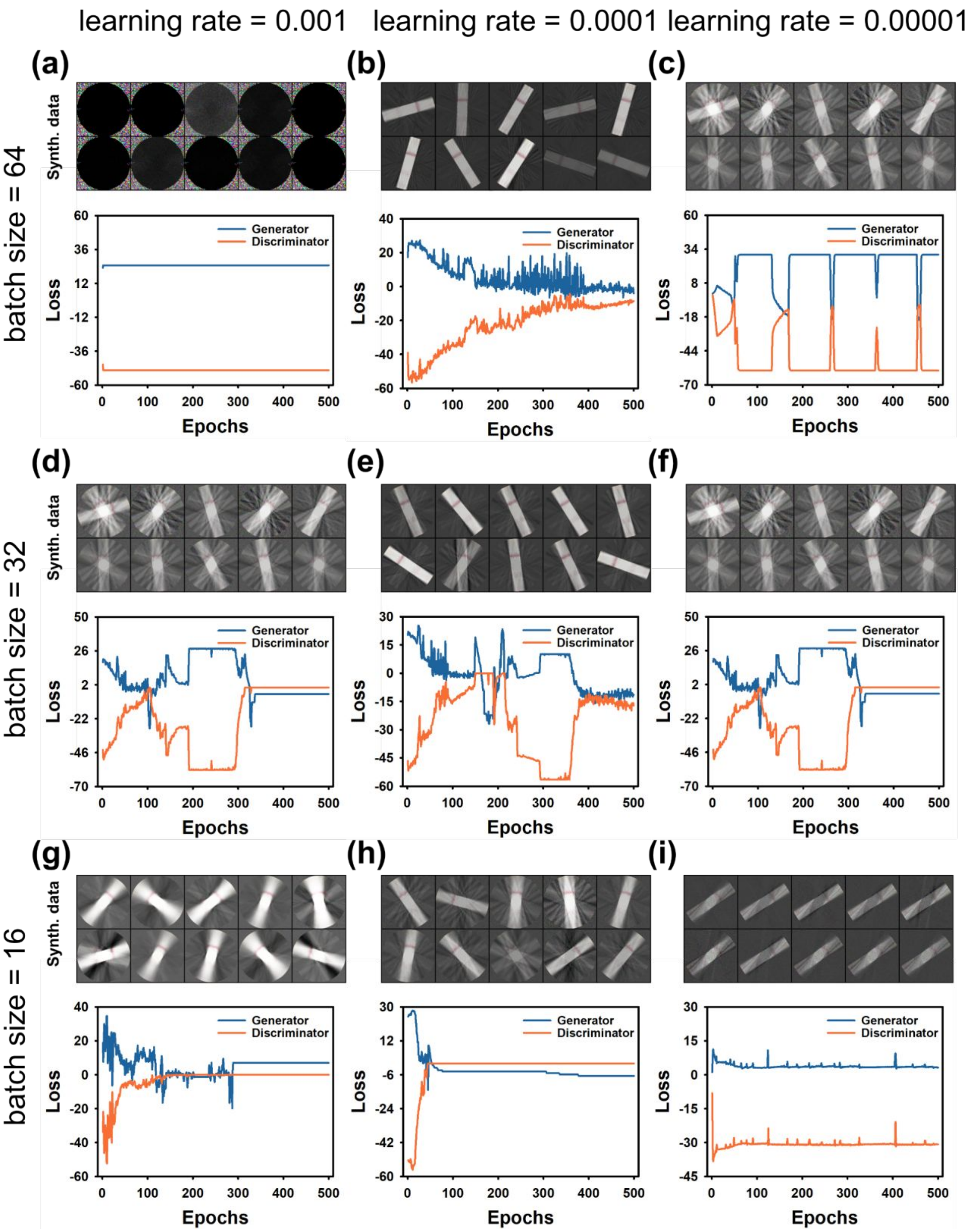

Fig. S6. Training stability analysis of SAGAN using the RMSprop optimizer, evaluated across different parameters: (a) learning rate = 0.001, batch size = 64; (b) learning rate = 0.0001, batch size = 64; (c) learning rate = 0.00001, batch size = 64; (d) learning rate = 0.001, batch size = 32; (e) learning rate = 0.0001, batch size = 32; (f) learning rate = 0.00001, batch size = 32; (g) learning rate = 0.001, batch size = 16; (h) learning rate = 0.0001, batch size = 16; (i) learning rate = 0.00001, batch size = 16.

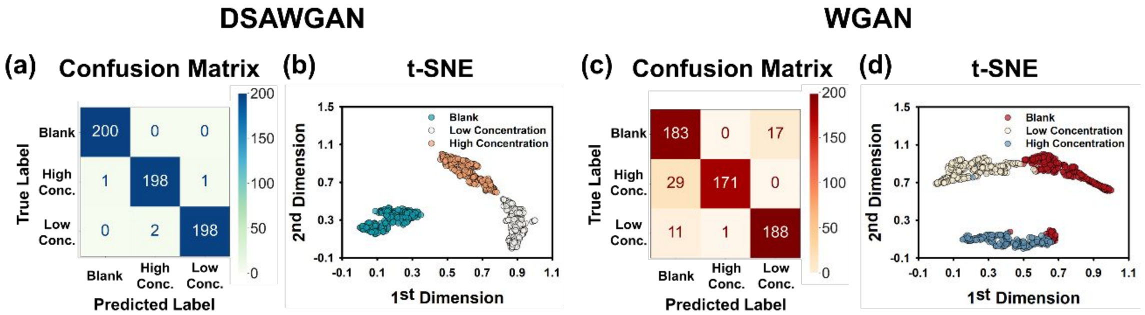

Fig. S7. Testing and comparison of experimental results for classification neural networks trained on different types of mixed datasets. (a–b) show the testing set classification results of networks trained on DSAWGAN mixed datasets, while (c–d) show those trained on WGAN mixed datasets.

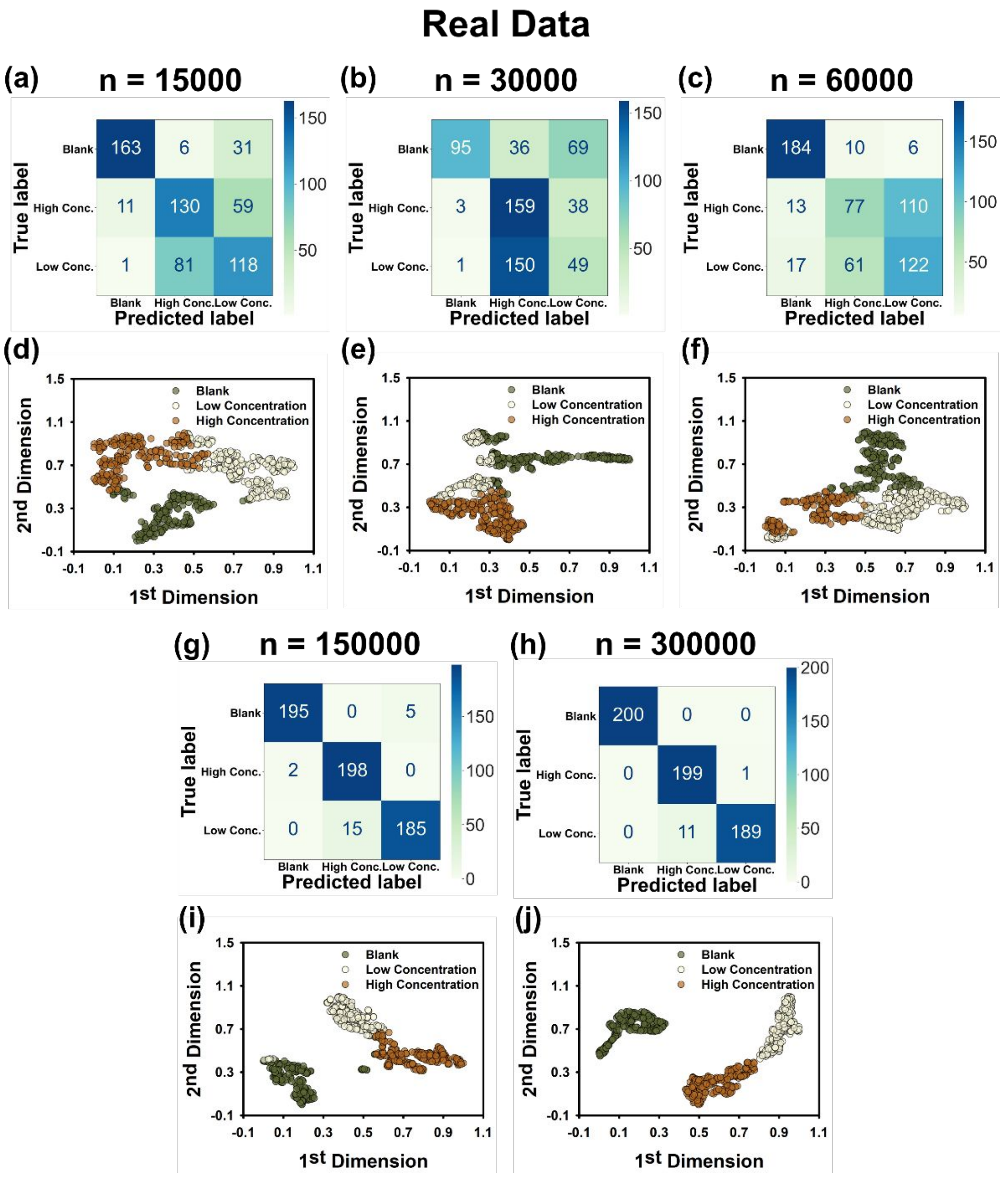

Fig. S8. Examination of the training results of classification neural networks using real dataset of different magnitudes, and analysis and visualization of the outcomes through confusion matrices and t-SNE plots. (**A-E**) Confusion matrices for testing set predictions of classification neural networks trained on datasets of different magnitudes. (**F-J**) t-SNE plots illustrating classification results on the testing set for datasets of different magnitudes.

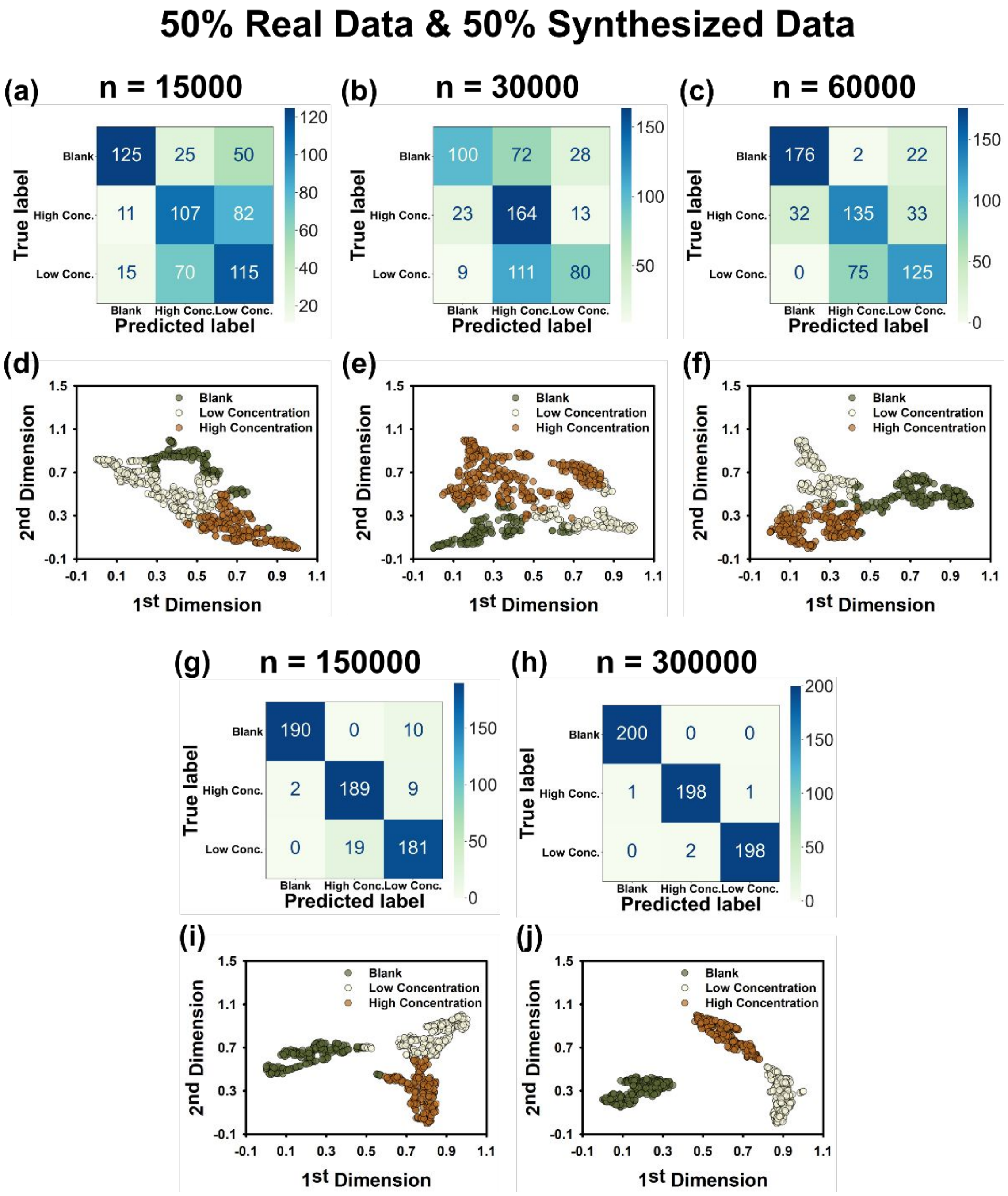

Fig. S9. Examination of the training results of classification neural networks using mixed dataset of different magnitudes, and analysis and visualization of the outcomes through confusion matrices and t-SNE plots. (**A-E**) Confusion matrices for testing set predictions of classification neural networks trained on datasets of different magnitudes. (**F-J**) t-SNE plots illustrating classification results on the testing set for datasets of different magnitudes.

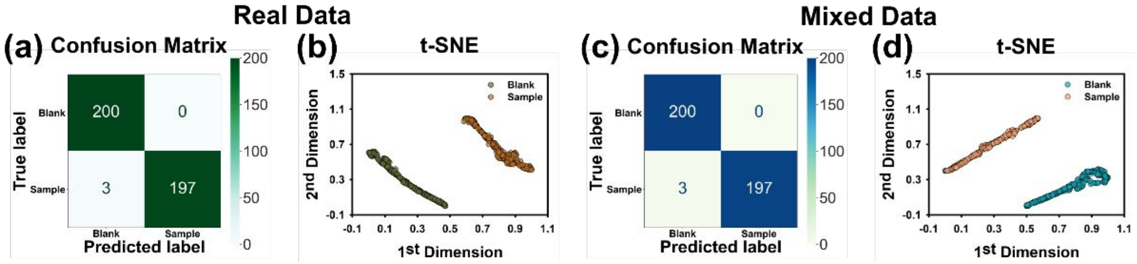

Fig. S10. Testing and comparison of the experimental results of training classification neural networks using real datasets and mixed datasets, applied to binary label data. (**a-b**) Represent the testing set classification results of classification neural networks trained on real datasets. (**c-d**) Represent the testing set classification results of classification neural networks trained on mixed datasets.

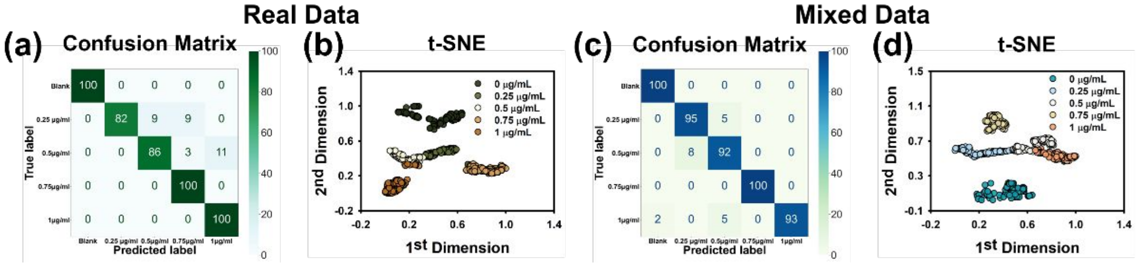

Fig. S11. Testing and comparison of the experimental results of training classification neural networks using real datasets and mixed datasets, applied to multi-label data. (**a-b**) Represent the testing set classification results of classification neural networks trained on real datasets. (**c-d**) Represent the testing set classification results of classification neural networks trained on mixed datasets.

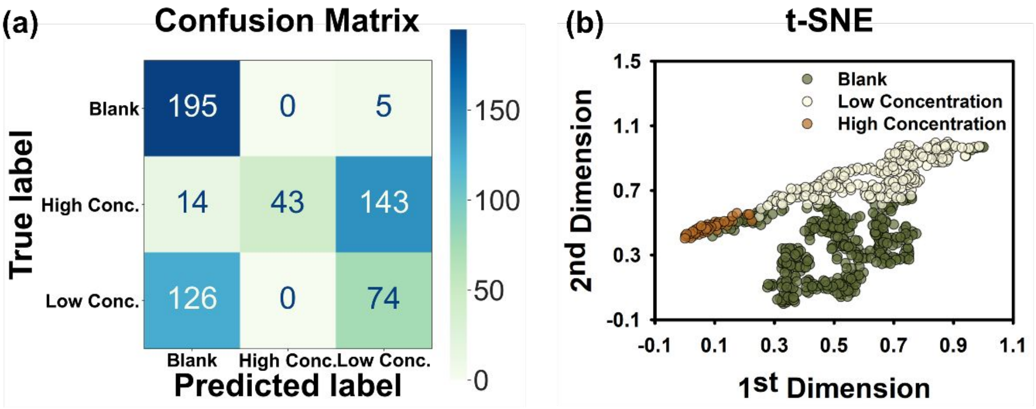

Fig. S12. Testing and comparison of the results of training classification neural networks using datasets formed by augmenting (flipping) real data, applied to the testing set. (**a**) Presents the training results using a confusion matrix. (**b**) Illustrates the training results using a t-SNE plot.

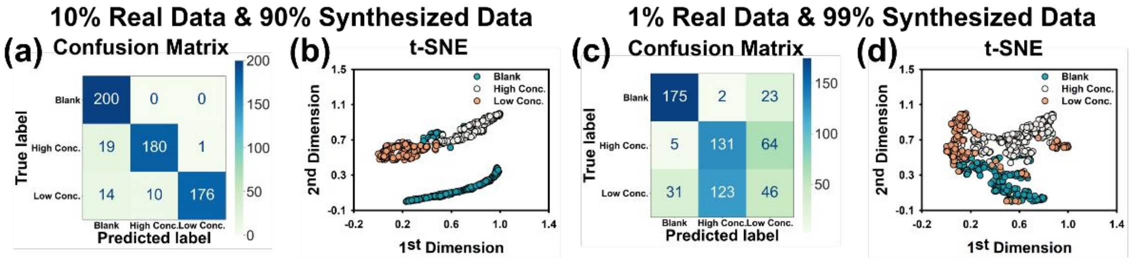

Figure S13. Testing and comparison of the experimental results of training classification neural networks using different ratios of mixed data. (**a-b**) Represent the experimental results of classifying the test set using a neural network trained with real data. (**c-d**) Represent the experimental results of classifying the test set using a neural network trained with mixed data.

Table S1. Composition of each classification in the training set, test set, and real-time detection data, including high concentration, low concentration, and negative data.

	High Conc.	Low Conc.	Blank	Total
Training Set	1000	1000	1000	3000
Test Set	200	200	200	600
Real-Time Analysis Data	10	10	10	30
Clinical Sample	10	10	10	30

Table S2. Comparison of training efficiency between DSAWGAN and other widely used GAN architectures, including DCGAN, SAGAN, and WGAN.

Model	DSAWGAN	SAGAN	WGAN	DCGAN
Training Time (sec.)	25512	27075	25054	12004

Table S3. Training results of classification neural networks using different types of mixed datasets, showing accuracy, sensitivity, and specificity for different labeled data.

	Accuracy	Blank		High Conc.		Low Conc.	
		Sensitivity	Specificity	Sensitivity	Specificity	Sensitivity	Specificity
DSAWGAN	0.9933	0.9975	1.0000	0.9950	0.9900	0.9975	0.9900
WGAN	0.9033	0.9000	0.9150	0.9975	0.8550	0.9575	0.9400

Table S4. Comparison of the training results of classification neural networks using datasets of different magnitudes, including real and mixed datasets.

Real Data							
**Amount of Data**	**Accuracy**	**Blank**		**High Conc.**		**Low Conc.**	
		**Sensitivity**	**Specificity**	**Sensitivity**	**Specificity**	**Sensitivity**	**Specificity**
**15000**	0.6900	0.9700	0.8150	0.7825	0.6500	0.7750	0.5900
**30000**	0.5100	0.9900	0.4750	0.5350	0.7950	0.7325	0.2450
**60000**	0.6400	0.9250	0.9200	0.8225	0.3850	0.7100	0.6100
**150000**	0.9600	0.9950	0.9750	0.9625	0.9900	0.9875	0.9250
**300000**	0.9800	1.0000	1.0000	0.9725	0.9950	0.9975	0.9450
**50% Real Data & 50% Synthesized Data**							
**Amount of Data**	**Accuracy**	**Blank**		**High Conc.**		**Low Conc.**	
		**Sensitivity**	**Specificity**	**Sensitivity**	**Specificity**	**Sensitivity**	**Specificity**
**15000**	0.5800	0.9350	0.6250	0.7625	0.5350	0.6700	0.5750
**30000**	0.5700	0.9200	0.5000	0.5425	0.8200	0.8975	0.4000
**60000**	0.7300	0.9200	0.8800	0.8075	0.6750	0.8625	0.6250
**150000**	0.9300	0.9950	0.9500	0.9525	0.9450	0.9525	0.9050
**300000**	0.9933	0.9975	1.0000	0.9950	0.9900	0.9975	0.9900

Table S5. Testing and comparison of classification neural networks trained on real and mixed datasets for binary label data. The results include accuracy, sensitivity, and specificity.

	Acc.	Blank		Sample	
		SEN	SPE	SEN	SPE
Real Data	0.9925	0.9850	1.0000	1.0000	0.9850
Mixed Data	0.9925	0.9850	1.0000	1.0000	0.9850

Table S6. Testing and comparison of classification neural networks trained on real and mixed datasets for multi-label data. The results include accuracy, sensitivity, and specificity.

		Real Data	Mixed Data
Accuracy		93.60%	96.00%
Blank	Sensitivity	100.00%	99.50%
	Specificity	100.00%	100.00%
0.25 µg/mL	Sensitivity	100.00%	98.00%
	Specificity	82.00%	95.00%
0.5 µg/mL	Sensitivity	97.75%	97.50%
	Specificity	86.00%	92.00%
0.75 µg/mL	Sensitivity	97.00%	100.00%
	Specificity	100.00%	100.00%
1 µg/mL	Sensitivity	97.25%	100.00%
	Specificity	100.00%	93.00%

Table S7. Testing and comparison of classification neural networks trained with datasets augmented by flipping real data, evaluated on the testing set. The results include accuracy, sensitivity, and specificity for different labeled data.

Acc.	Blank		High Conc.		Low Conc.	
	SEN	SPE	SEN	SPE	SEN	SPE
0.5200	0.6500	0.9750	1.0000	0.2150	0.6300	0.3700

Table S8. Training results of classification neural networks using different ratios of mixed datasets, showing accuracy, sensitivity, and specificity for various labeled data.

**10% Real Data & 90% Synthesized Data**						
Acc.	Blank		High Conc.		Low Conc.	
	SEN	SPE	SEN	SPE	SEN	SPE
0.9267	0.9175	1.0000	0.9750	0.9000	0.9975	0.880
**1% Real Data & 99% Synthesized Data**						
Acc.	Blank		High Conc.		Low Conc.	
	SEN	SPE	SEN	SEN	SPE	SEN
0.5867	0.9100	0.8750	0.6875	0.6550	0.7825	0.2300

Table S9. Classification results for real-time detection data on the Sony Z5.

	True Label	High Conc.	Low Conc.	Blank	Predict Label
**#1**	**High Conc.**	0.5120	0.1480	0.3400	**High Conc.**
**#2**	**High Conc.**	0.9730	0.0270	0.0000	**High Conc.**
**#3**	**High Conc.**	0.8130	0.0470	0.1400	**High Conc.**
**#4**	**High Conc.**	0.7810	0.1060	0.1130	**High Conc.**
**#5**	**High Conc.**	0.6760	0.2300	0.0940	**High Conc.**
**#6**	**High Conc.**	0.8240	0.1170	0.0590	**High Conc.**
**#7**	**High Conc.**	0.6840	0.3120	0.0040	**High Conc.**
**#8**	**High Conc.**	0.4140	0.2500	0.3360	**High Conc.**
**#9**	**High Conc.**	0.7730	0.1680	0.0590	**High Conc.**
**#10**	**High Conc.**	0.5080	0.1090	0.3830	**High Conc.**
**#11**	**Low Conc.**	0.0820	0.7660	0.1520	**Low Conc.**
**#12**	**Low Conc.**	0.2340	0.5200	0.2460	**Low Conc.**
**#13**	**Low Conc.**	0.0000	0.6410	0.3590	**Low Conc.**
**#14**	**Low Conc.**	0.0000	0.8160	0.1840	**Low Conc.**
**#15**	**Low Conc.**	0.0510	0.9020	0.0470	**Low Conc.**
**#16**	**Low Conc.**	0.2100	0.6450	0.1450	**Low Conc.**
**#17**	**Low Conc.**	0.3910	0.5000	0.1090	**Low Conc.**
**#18**	**Low Conc.**	0.5120	0.3550	0.1330	**High Conc.**
**#19**	**Low Conc.**	0.3710	0.3400	0.2890	**High Conc.**
**#20**	**Low Conc.**	0.2340	0.5200	0.2460	**Low Conc.**
**#21**	**Blank**	0.0000	0.0160	0.9840	**Blank**
**#22**	**Blank**	0.0310	0.1210	0.8480	**Blank**
**#23**	**Blank**	0.0430	0.1600	0.7970	**Blank**
**#24**	**Blank**	0.0820	0.0590	0.8590	**Blank**
**#25**	**Blank**	0.0000	0.0350	0.9650	**Blank**
**#26**	**Blank**	0.0000	0.0040	0.9960	**Blank**
**#27**	**Blank**	0.0000	0.0230	0.9770	**Blank**
**#28**	**Blank**	0.0310	0.0120	0.9570	**Blank**
**#29**	**Blank**	0.0120	0.0350	0.9530	**Blank**
**#30**	**Blank**	0.0040	0.0040	0.9920	**Blank**

Table S10. Classification results for real-time detection data on the Redmi Note 6 Pro.

	True Label	High Conc.	Low Conc.	Blank	Predict Label
**#1**	**High Conc.**	0.8980	0.0900	0.0120	**High Conc.**
**#2**	**High Conc.**	0.7580	0.2030	0.0390	**High Conc.**
**#3**	**High Conc.**	0.8750	0.0980	0.0270	**High Conc.**
**#4**	**High Conc.**	0.8670	0.1090	0.0240	**High Conc.**
**#5**	**High Conc.**	0.7110	0.2420	0.0470	**High Conc.**
**#6**	**High Conc.**	0.8980	0.0590	0.0430	**High Conc.**
**#7**	**High Conc.**	0.8440	0.0940	0.0620	**High Conc.**
**#8**	**High Conc.**	0.6520	0.2500	0.0980	**High Conc.**
**#9**	**High Conc.**	0.8790	0.0860	0.0350	**High Conc.**
**#10**	**High Conc.**	0.8160	0.0900	0.0940	**High Conc.**
**#11**	**Low Conc.**	0.0550	0.8440	0.1010	**Low Conc.**
**#12**	**Low Conc.**	0.0350	0.7700	0.1950	**Low Conc.**
**#13**	**Low Conc.**	0.0860	0.7970	0.1170	**Low Conc.**
**#14**	**Low Conc.**	0.0160	0.7420	0.2420	**Low Conc.**
**#15**	**Low Conc.**	0.0080	0.6370	0.3550	**Low Conc.**
**#16**	**Low Conc.**	0.0120	0.6130	0.3750	**Low Conc.**
**#17**	**Low Conc.**	0.0080	0.8950	0.0970	**Low Conc.**
**#18**	**Low Conc.**	0.0280	0.9450	0.0270	**Low Conc.**
**#19**	**Low Conc.**	0.0660	0.6410	0.2930	**Low Conc.**
**#20**	**Low Conc.**	0.0000	0.7380	0.2620	**Low Conc.**
**#21**	**Blank**	0.0550	0.1950	0.7500	**Blank**
**#22**	**Blank**	0.0000	0.0700	0.9300	**Blank**
**#23**	**Blank**	0.0120	0.1520	0.8360	**Blank**
**#24**	**Blank**	0.0900	0.2300	0.6800	**Blank**
**#25**	**Blank**	0.0000	0.0120	0.9880	**Blank**
**#26**	**Blank**	0.0000	0.0230	0.9770	**Blank**
**#27**	**Blank**	0.0040	0.0120	0.9840	**Blank**
**#28**	**Blank**	0.0000	0.0040	0.9960	**Blank**
**#29**	**Blank**	0.0000	0.0080	0.9920	**Blank**
**#30**	**Blank**	0.0080	0.0040	0.9880	**Blank**

Table S11. Classification results for real-time detection data on the Asus Zenfone 5Z.

	True Label	High Conc.	Low Conc.	Blank	Predict Label
**#1**	**High Conc.**	0.9410	0.0550	0.0040	**High Conc.**
**#2**	**High Conc.**	0.9770	0.0200	0.0030	**High Conc.**
**#3**	**High Conc.**	0.9650	0.0190	0.0160	**High Conc.**
**#4**	**High Conc.**	0.9960	0.0040	0.0000	**High Conc.**
**#5**	**High Conc.**	0.9800	0.0200	0.0000	**High Conc.**
**#6**	**High Conc.**	0.9410	0.0590	0.0000	**High Conc.**
**#7**	**High Conc.**	0.8520	0.1170	0.0310	**High Conc.**
**#8**	**High Conc.**	0.9020	0.0780	0.0200	**High Conc.**
**#9**	**High Conc.**	0.9300	0.0550	0.0150	**High Conc.**
**#10**	**High Conc.**	0.9530	0.0430	0.0040	**High Conc.**
**#11**	**Low Conc.**	0.0320	0.9410	0.0270	**Low Conc.**
**#12**	**Low Conc.**	0.0230	0.9730	0.0040	**Low Conc.**
**#13**	**Low Conc.**	0.0130	0.9770	0.0100	**Low Conc.**
**#14**	**Low Conc.**	0.0230	0.9610	0.0160	**Low Conc.**
**#15**	**Low Conc.**	0.0160	0.9690	0.0150	**Low Conc.**
**#16**	**Low Conc.**	0.0040	0.9800	0.0160	**Low Conc.**
**#17**	**Low Conc.**	0.0200	0.9600	0.0200	**Low Conc.**
**#18**	**Low Conc.**	0.0460	0.9380	0.0160	**Low Conc.**
**#19**	**Low Conc.**	0.0000	0.9730	0.0270	**Low Conc.**
**#20**	**Low Conc.**	0.0120	0.9650	0.0230	**Low Conc.**
**#21**	**Blank**	0.0000	0.0350	0.9650	**Blank**
**#22**	**Blank**	0.0000	0.0270	0.9730	**Blank**
**#23**	**Blank**	0.0160	0.0430	0.9410	**Blank**
**#24**	**Blank**	0.0120	0.2500	0.7380	**Blank**
**#25**	**Blank**	0.0270	0.1290	0.8440	**Blank**
**#26**	**Blank**	0.0000	0.0860	0.9140	**Blank**
**#27**	**Blank**	0.0000	0.0080	0.9920	**Blank**
**#28**	**Blank**	0.0000	0.0040	0.9960	**Blank**
**#29**	**Blank**	0.0000	0.0120	0.9880	**Blank**
**#30**	**Blank**	0.0080	0.0470	0.9450	**Blank**
